# Psychosocial support for schoolchildren in wartime Ukraine: community-based access and parent-reported perceived helpfulness

**DOI:** 10.1186/s13031-026-00762-9

**Published:** 2026-02-08

**Authors:** Olena Yelizarova, Tetiana Stankevych, Alla Parats, Viktor Yelizarov, Olha Puzanova, Natalia Lebedynets, Svitlana Hozak

**Affiliations:** 1 State Institution Marzieiev Institute for Public Health of the National Academy of Medical Sciences of Ukraine, 50 Hetman Pavlo Polubotok Street, Kyiv, 02094 Ukraine; 2 Luxoft Ukraine, 10\14 Volnovaska Street, Kyiv, 03124 Ukraine; 3 PHEI “Kyiv Medical University”, 2 Boryspilska Street, Kyiv, 02099 Ukraine; 4 Dragomanov Ukrainian State University, 9 Pyrogova Street, Kyiv, 02000 Ukraine; 5 National University of Ukraine of Physical Education and Sport, 1 Fizkultury Street, Kyiv, 02000 Ukraine

**Keywords:** MHPSS, Schoolchildren, Ukraine, War, Community-based support, Access to care, Parent-reported perceived helpfulness, Social support

## Abstract

**Background:**

Since the onset of the full-scale war in Ukraine in 2022, numerous initiatives have been launched to strengthen mental health and psychosocial support (MHPSS), particularly for children and adolescents. Community-based psychosocial support has been declared a national priority; however, the availability, accessibility, and perceived helpfulness of such support remain insufficiently documented at the population level under conditions of ongoing conflict.

**Objective:**

This study aimed to characterize access to psychosocial support among Ukrainian schoolchildren during wartime and to assess parent-reported perceived helpfulness of received support, with particular attention to social and community factors shaping help-seeking and perceived benefit.

**Methods:**

Between 2022 and 2025, a nationwide survey was conducted among parents and caregivers of school-aged children (*N* = 7,551). Indicators were introduced iteratively across survey waves in response to evolving wartime conditions; analyses for each outcome were restricted to the years in which the respective item was measured. Binary logistic regression models were used to examine associations between sociodemographic, psychosocial, and contextual factors and (2022–2025) help-seeking and (2023–2025) perceived helpfulness.

**Results:**

Overall, 12.0% of parents reported that their children needed psychological support, while 9.9% reported contacting a psychologist. Among those who accessed services, 68.1% rated the received support as helpful. Perceived helpfulness was lower among children with anxiety and depressive symptoms (53.9%). The presence of potential anxiety and depressive symptoms in children was the strongest predictor of help-seeking (OR = 3.31; 95% CI: 2.69–4.09) and was also associated with lower perceived helpfulness (OR = 0.55; 95% CI: 0.37–0.81). In contrast, parental anxiety was independently associated with increased help-seeking (OR = 1.24; 95% CI: 1.01–1.53), but not with perceived helpfulness. Urban residence and displacement were positively associated with service use, whereas rural residence and lack of migration experience were associated with lower access. While parental education and general social support were not significant predictors of help-seeking, higher levels of social support were associated with greater perceived helpfulness, highlighting the role of interpersonal and community resources. Parent-reported perceived helpfulness appears to be shaped not only by children’s mental health needs but also by parental well-being and broader social context. Based on these findings, a multisectoral model was developed to improve identification, referral, and support pathways for children in crisis.

**Conclusions:**

Substantial gaps persist between perceived need and access to psychosocial support for Ukrainian schoolchildren during wartime, particularly in rural settings. Strengthening community-based psychosocial services, improving outreach, and supporting families with limited resources may help reduce inequalities in access to and perceived benefits of MHPSS during prolonged conflict.

**Supplementary Information:**

The online version contains supplementary material available at 10.1186/s13031-026-00762-9.

## Background

Psychosocial assistance during emergencies constitutes a set of essential measures aimed at mitigating health risks and strengthening the resilience of individuals, communities, and public systems [1]. Such support is required in contexts affected by natural hazards, technological disasters, or armed conflict, where populations face not only physical harm but also profound psychological strain necessitating timely and accessible interventions [2, 3]. Timely psychosocial support can reduce both immediate and long-term mental health consequences, underscoring the need for coordinated multisectoral efforts involving governmental, non-governmental, and public health actors [4, 5].

Exposure to severe and chronic stressors, such as war, terrorism, violence, displacement, and breakdown of community support networks, increases the likelihood of depression, post-traumatic stress disorder, anxiety, sleep disturbances, and functional somatic symptoms across all age groups, including children and adolescents [6–8]. In such settings, communities require an integrated system that includes informal support networks, primary healthcare providers, and specialized mental health services [7, 9].

In Ukraine, the Law “On the Public Health System” (2573-IX, 2024) identifies community-level psychosocial support as a core component of the national public health strategy [10]. This legislation aims to strengthen mental health promotion, prevent mental disorders, and improve access to psychological services. However, the development of a comprehensive and functional psychosocial support system remains at an early stage and requires consistent monitoring and evaluation to guide implementation [11].

Community-based psychosocial support strategies commonly involve awareness-raising, psychoeducation, stigma reduction campaigns, and initiatives to facilitate access to mental health services and resources [1, 4, 12]. Effective planning of such interventions requires population-based data on mental health status, help-seeking behaviors, and contextual factors influencing access to care. The integration of social determinants of health into program design is essential for evaluating equity, feasibility, and real-world effectiveness [12].

Since 2022, multiple interventions have been introduced in Ukraine through the Mental Health and Psychosocial Support System (MHPSS), including hotlines, community support groups, and school-based programs employing psychological tools and short-term interventions tailored to various population groups [11–19]. Children and adolescents represent a priority population, as developmental sensitivity to stressors heightens their vulnerability and increases the importance of early detection, prevention, and sustained monitoring [13].

From a public health standpoint, parent-reported perceived helpfulness represents a key experiential outcome that is particularly relevant in settings characterized by ongoing exposure, limited clinical follow-up, and persistent stigma surrounding mental healthcare [20]. Such perceptions can serve as a proxy for the acceptability, feasibility, and contextual fit of psychosocial interventions, complementing traditional symptom-based outcomes [21–23]. This metric is increasingly used in low-resource and conflict-affected settings as a pragmatic indicator of alignment with family needs and real-world service responsiveness.

Community-based psychosocial support encompasses a broad continuum of non-clinical activities, including psychoeducation, skills training, peer-support initiatives, and school-based engagement [22]. These components are crucial for accessibility and sustained engagement, especially where formal services remain limited and community networks serve as primary support structures, as evidenced by global meta-reviews of community mental health platforms [23].

Taken together, these considerations underscore the need for population-based evidence on how psychosocial support for children is accessed and experienced during wartime. This study addresses this gap by examining community-based help-seeking patterns and assessing parental perceptions of support helpfulness as an indicator of responsiveness, accessibility, and contextual relevance across social and demographic strata.

## Methods

### Aim

The study aimed to characterize community-based access to psychosocial support among Ukrainian schoolchildren during wartime and to assess the parent-reported perceived helpfulness of the support received.

### Study design

The study consisted of repeated cross-sectional online survey waves conducted annually between 2022 and 2025. Each wave included independent respondents; no individual-level follow-up was performed. Data were collected using the Q-RAPH questionnaire, initially developed during the COVID-19 pandemic and adapted for wartime conditions [24]. The survey comprised closed-ended items and optional open-text fields for parental comments.

Ethical approval was obtained from the institutional bioethics committee (Protocol No. 1, February 28, 2022). Parents were informed about the sensitive nature of mental health–related items, and survey links provided information on psychosocial support resources. Participation was voluntary and anonymous. Parents could optionally leave contact details if they wished to receive guidance; in such cases, the corresponding author, a medical psychologist, contacted them to provide supportive information.

Additional methodological justification related to the online mode, digitalization context, and parental engagement is provided in Appendix A.

## Participants and recruitment procedures

The target population comprised parents or primary caregivers of school-aged children (8–18 years), residing in Ukraine or abroad at the time of participation.

Across all study years, data collection was conducted exclusively online. While the survey mode remained consistent, recruitment channels and sampling frames differed across waves in response to contextual constraints.

In 2022, during periods of acute insecurity and widespread school disruption, recruitment relied on voluntary online snowball sampling. The survey link was disseminated via institutional websites, social media platforms, and informal parent networks. Parents frequently shared the link within online parent communities, reflecting prevailing communication practices under crisis conditions.

From 2023 onward, a stratified random selection of public (municipal) schools was conducted using national registries of the Ministry of Education and Science. Schools were stratified by oblast. Within each region, schools were assigned numeric identifiers and randomly selected via an online tool. Very small schools were replaced to ensure feasibility. Regional Departments of Education verified eligibility and facilitated distribution.

Survey links were distributed by schools through established digital channels used for routine school–parent communication. The recruitment target was approximately 1,000 responses per region. Based on prior online survey response rates, links were disseminated to an estimated 15,000 parents per region to meet this goal.

No financial or material incentives were offered in any wave.

## Sampling considerations and weights

Because recruitment strategies differed across waves and data collection was exclusively online, the sample was not fully probability-based. To improve alignment with national school population characteristics, post-stratification weights were applied for each survey year based on official MoE data. Weighting variables included urban/rural residence, child sex, and educational stage (primary, secondary, high).

Weighted estimates were used for descriptive sensitivity analyses, while regression models were conducted using unweighted data to avoid inflated variance. Weighted and unweighted results are reported where appropriate.

## Sample formation and missing data

In total, 10,270 responses were received, of which 10,011 met inclusion criteria. Respondents were excluded if the child was younger than 8 years (*n* = 1,042), undergoing medical treatment or rehabilitation (*n* = 565), or had missing values on key variables. Complete-case analysis was used for regression models; no imputation was applied. Variables introduced in later waves were analysed only for the relevant years.

**Fig. 1:**
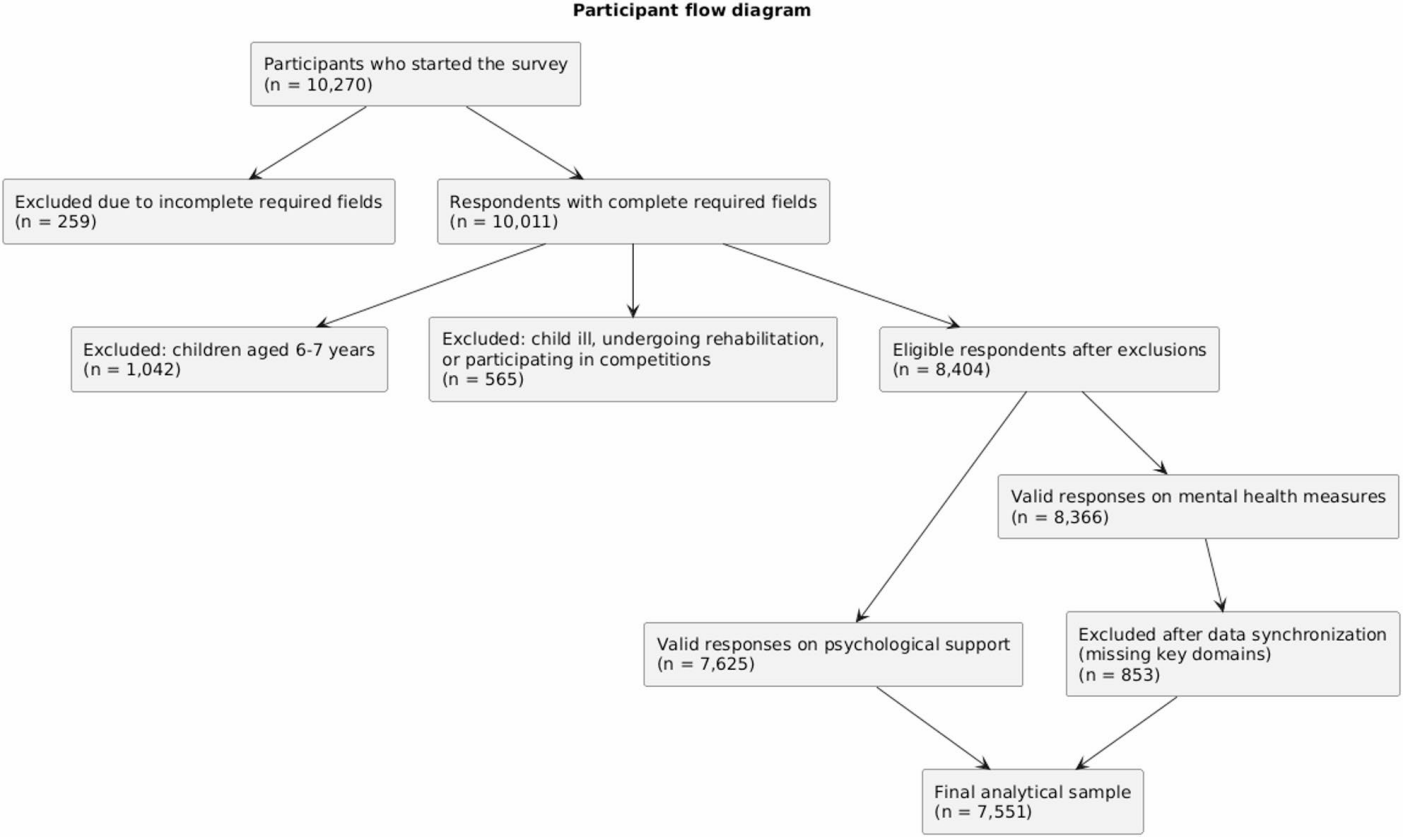
Flow diagram of sample formation with exclusions and final analytical sample (*n* = 7,551)

The final analytical sample consisted of 7,551 respondents. A flow diagram is presented in Fig. 1.

## Measures

### Mental health assessment

Children’s mental health symptoms were measured using the RCADS-P-25 (Revised Child Anxiety and Depression Scale – Parent Version), a validated screening instrument widely used in epidemiological and public health research [25–27]. The scale aligns with IASC recommendations for psychosocial assessment in humanitarian settings [28]. The Ukrainian version was culturally adapted using a standard forward–backward translation procedure and was approved by the original scale developers.

Importantly, the RCADS-P-25 was used in this study as a screening tool, not a diagnostic instrument. No clinical interviews were conducted, and symptom scores were not interpreted as clinical diagnoses. Instead, the scale served as an indicator of elevated vulnerability and psychosocial risk, in line with its recommended use in large-scale epidemiological and public health research.

Parental report was the primary source of information on children’s mental health. Although parent assessments may have limited sensitivity to subtle internal experiences, previous research indicates acceptable concordance between parent and child reports for structured symptom questionnaires, particularly for salient emotional and behavioral indicators. In this study, parents were explicitly encouraged to consult with their child when answering items about emotional well-being and perceived psychosocial support.

Symptom severity was operationalized using standardized T-scores, with values ≥ 65 indicating elevated anxiety or depressive symptoms according to the original scoring framework. The psychometric properties of the Ukrainian version of the RCADS-P-25, including reliability estimates across study years and age bands, are detailed in Supplementary Appendix C. Across aggregated data from 2022 to 2025, composite reliability coefficients were: ω = 0.79 (Anxiety), ω = 0.81 (Depression); SEM-based α = 0.80 (Anxiety), α = 0.81 (Depression); Ordinal α = 0.93 for both scales. Test–retest reliability was high, with intraclass correlation coefficients (ICC) ranging from 0.92 to 0.96.

Parental symptoms of depression and anxiety were assessed using the PHQ-2 and GAD-2 screeners. Due to the two-item format of these scales, internal consistency was assessed using inter-item Spearman correlations (rather than traditional α coefficients), which demonstrated good consistency: PHQ-2: ρ = 0.60 (α = 0.75), GAD-2: ρ = 0.54 (α = 0.70).

Test–retest reliability was assessed in a subsample using ICCs (two-way mixed effects, consistency), showing moderate-to-good reliability for single measures (ICC = 0.58–0.66) and good-to-excellent reliability for average measures (ICC = 0.84–0.89).

## Access and perception of psychological support

Four indicators were used:

1. Help-seeking behaviour.
2. Perceived helpfulness of psychological support.
3. Barriers to accessing support.
4. Perceived social support (informal and state-provided).

Table 1 summarizes item wording and years of availability.

Receipt of support was inferred through skip logic: parents who reported seeking help subsequently evaluated its helpfulness.

Open-text responses were classified using inductive thematic coding by two co-authors (Appendix D). Coding was intended for descriptive illustration; no quantitative reliability testing was performed.

Social support was measured via two items assessing perceived informal (family, friends, volunteers) and state-provided support on 5-point scales.

**Table 1:** Overview of MHPSS indicators: sources and year of inclusion

Indicator	Questionnaire item	Years available
Perceived need for psychological help	“Did your child need psychological help during the war?” *(Yes/No)*	2023–2025
Help-seeking behavior	“Did you seek psychological help for your child?” *(Yes/No)*	2022–2025
Perceived helpfulness of psychosocial support (parent-reported)	“Was psychological help helpful?” *(Yes/No)*	2023–2025
Barriers to accessing psychological support	“If you did not seek help, what prevented you?” *(Open-ended response)*	2023–2025
School-based interventions	“Are ‘psychological minutes’ conducted at your child’s school?” *(Yes/No)*	2022–2025
Community-based social support	“Do you feel supported by your social circle (friends, family, volunteers)?” *(1 to 5 scale)*	2022–2025
State-provided social support	“Do you feel supported by the state (local authorities, social services)?” *(1 to 5 scale)*	2024–2025

The analytic cascade presented in this study follows four sequential stages derived from the available indicators

Perceived need → Sought psychological help → Received support (inferred) → Perceived helpfulness

“Receipt of support” was not assessed through a standalone item but was inferred based on skip logic: all respondents who reported seeking help subsequently answered the perceived helpfulness item, indicating contact with a provider

Years reflect availability of each indicator in successive survey waves. Some items (e.g., state support) were added in later iterations to capture evolving contextual dimensions of MHPSS under wartime conditions

Summary of mental health and psychosocial support (MHPSS) indicators included in the Q-RAPH questionnaire, with respective data sources and years of collection

**Fig. 2:**
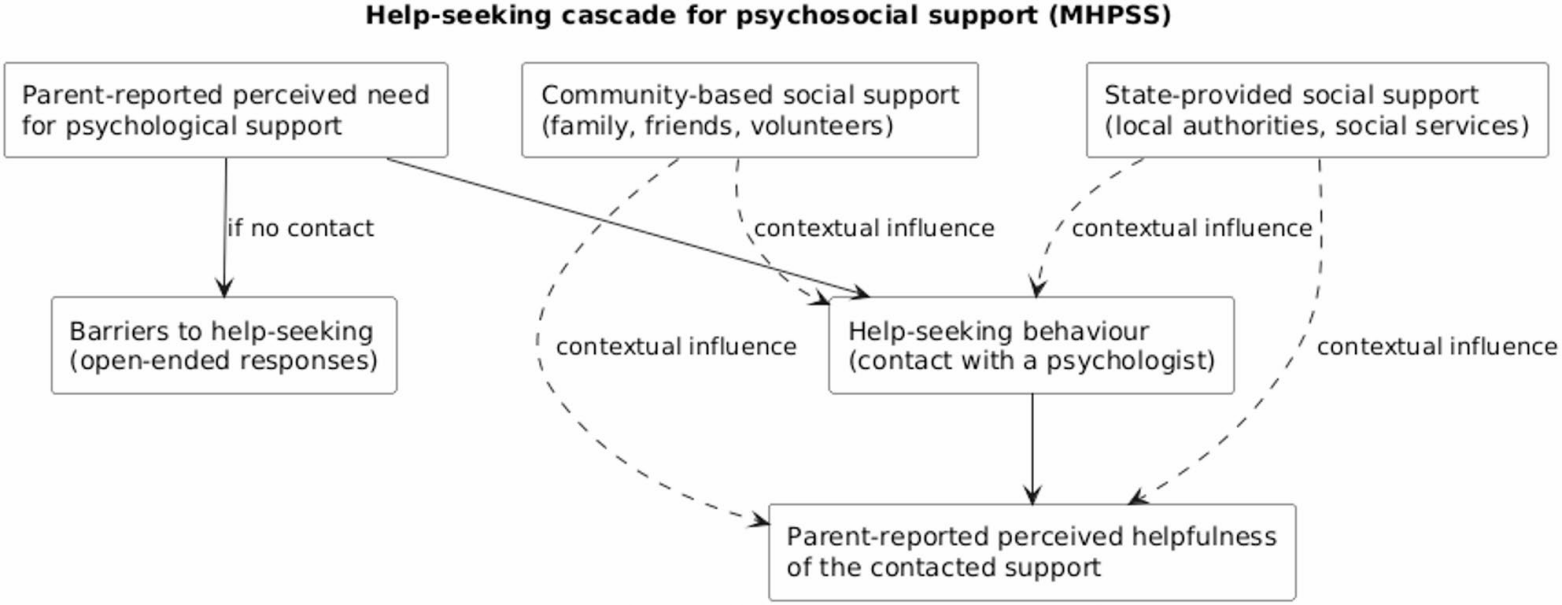
Conceptual framework of help-seeking and perceived helpfulness of psychosocial support. The figure illustrates the assessment logic of psychosocial support, including parent-reported perceived need for psychological support, help-seeking behaviour (contact with a mental health professional), and parent-reported perceived helpfulness of the contacted support. Social support is conceptualized as a contextual factor influencing help-seeking and perceived helpfulness (dotted lines)

Figure 2 presents the conceptual cascade: Perceived need → Help-seeking → Receipt of support (inferred) → Perceived helpfulness. This approach is consistent with monitoring frameworks in humanitarian MHPSS evaluation [5].

The framework emphasizes that the principal loss in the pathway typically occurs before help-seeking—during need recognition and decision-making—rather than at the level of service availability.

The framework emphasizes that the primary loss in the support pathway occurs before help-seeking, at the stage of need recognition and decision-making, rather than due to lack of available services.

This combination of quantitative and qualitative indicators allows for a multidimensional assessment of the mental health and psychosocial support system, while also acknowledging contextual variability across the wartime timeline.

The symptom–need interpretation gap (SNIG) was defined as the discrepancy between elevated symptoms on the RCADS-P-25 and the absence of parent-reported perceived need for psychological support. Elevated symptoms do not imply diagnosis; rather, they indicate increased vulnerability.

### AI-assisted support

AI-assisted support (GPT 4o, 5.1, 5.2, OpenAI) was used as part of an interactive revision process to refine terminology and strengthen conceptual clarity. During manuscript revision, AI-assisted support was used to improve coherence of argumentation and to support consistency between empirical results and conceptual figures. The study design, data analyses, and interpretation of results were conducted entirely by the authors.

### Statistical analysis

Descriptive statistics were calculated for all variables. Group differences were tested using Pearson’s χ² or likelihood-ratio χ² when cell counts were < 10.

Binary logistic regression models were used to examine predictors of:

5. Help-seeking behaviour.
6. Perceived helpfulness of psychological support.

Model fit was assessed using likelihood-ratio tests, pseudo-R², Hosmer–Lemeshow tests, and ROC-based discrimination. Robustness checks using post-stratification weights and exploratory interactions are reported.

Independent variables included: child age, sex, BMI (WHO standards), chronic illness, urban–rural residence, region, migration status, parental age and education, anxiety/depression symptoms, and perceived social support. VIF values were < 2.0, indicating no multicollinearity (Appendix F).

Analyses were performed in RStudio and SPSS 26.

## Results

The final analytical sample included 7,551 parents of school-age children. The gender distribution was balanced (50.9% boys), and the mean child age was 11.6 years (SD = 2.7). The mean parental age was 39.3 years (SD = 6.0). Sample characteristics are presented in Table 2.

Overall, 35.2% of children were reported to have chronic illnesses, and 27.2% were classified as overweight or obese. Most participants resided in urban areas (76.7%). Regarding migration, 63.1% of families were non-displaced, 22.9% had returned after temporary displacement, 10.9% were internally displaced persons (IDPs), and 3.1% were residing abroad at the time of the survey.

**Table 2:** Sample characteristics

Categories	Sex				Total		χ^2^	*p*
	Boys		Girls					
	*n*	%	*n*	%	*n*	%		
**Year**:								
2022	250	49,0	260	51,0	510	6,8	0,8	0,837
2023	764	51,0	734	49,0	1498	19,8		
2024	701	51,1	670	48,9	1371	18,2		
2025	2127	51,0	2045	49,0	4172	55,3		
2022–2025	3842	50,9	3709	49,1	7551	100,0		
**Age group**:								
Children aged 8 to 11 years	2031	51,6	1902	48,4	3933	52,1	1,8	0.169
Adolescents aged 12 to 18 years	1811	50,1	1807	49,9	3618	47,9		
**BMI**								
underweight	129	43,7	166	56,3	295	3,9	52.8	0.001
normal	2529	48,7	2668	51,3	5197	68,9		
overweight and obesity	1180	57,6	870	42,4	2050	27,2		
**Chronic Diseases**:								
No	2442	50,1	2436	49,9	4878	64,8	3.4	0.066
Yes	1387	52,3	1266	47,7	2653	35,2		
**Migration**:								
Non-displaced	2468	51,8	2301	48,2	4769	63,2	4.5	0.209
Returned	852	49,3	875	50,7	1727	22,9		
Abroad^1^	110	47,2	123	52,8	233	3,1		
IDPs^2^	412	50,1	410	49,9	822	10,9		
**Place of residence**:								
Urban	2949	51,0	2839	49,1	5788	76,7	0.03	0.860
Rural	892	50,7	867	49,3	1759	23,3		
**Parental education levels**:								
Higher education	2599	51,0	2494	49,0	5093	67,5	0.5	0.798
Vocational education	853	50,2	845	49,8	1698	22,5		
General secondary education	389	51,5	366	48,5	755	10,0		

^1^Austria, Bulgaria, Great Britain, Georgia, Denmark, Estonia, Israel, Ireland, Spain, Italy, Kazakhstan, Canada, Kuwait, Latvia, Lithuania, Moldova, Netherlands, Germany, Norway, Poland, Portugal, Romania, Slovakia, USA, Turkey, Hungary, Finland, France, Croatia, Czech Republic, Montenegro, Switzerland, Sweden

^2^ IDPs are Internally Displaced Persons

Across 2022–2025, 12.8% of children screened positive for elevated anxiety or depressive symptoms (RCADS-P-25 T-score ≥ 65). Prevalence declined from 24.7% in 2022 to 9.4% in 2025 (χ² = 164.4; *p* < 0.001). Higher prevalence was observed among children with chronic illnesses (20.7%) and among displaced families (19.6%). Urban children had higher rates compared with rural peers (13.8% vs. 9.4%).

No significant sex differences were found in the overall sample (*p* > 0.4). Age-stratified analyses revealed higher prevalence among younger boys compared with younger girls (13.2% vs. 10.7%; *p* = 0.015), whereas among older adolescents, girls had higher rates than boys (17.9% vs. 11.1%; *p* = 0.012).

In the 2025 wave, 23.4% of parents reported symptoms of anxiety and 28.1% reported symptoms of depression. Across all waves, cumulative parental symptom prevalence was 27.0% (anxiety) and 31.5% (depression).

### Psychological support

Among children with elevated anxiety–depressive symptoms, 21.9 ± 1.4% sought psychological support, compared with 7.7 ± 0.3% among those without such symptoms (χ²=280.2; *p* < 0.001). Perceived helpfulness was reported by 53.9 ± 3.4% of parents whose children had elevated symptoms and 74.6 ± 2.0% of parents whose children did not (χ²=29.9; *p* < 0.001). Patterns were consistent across sex and age groups (Appendix D, Table D2).

Between 2023 and 2025, 12.1% of parents reported that their child had needed psychological support. Reported need varied significantly by chronic illness status (χ²=129.9), sex (χ²=87.6), age group (χ²=4.7), migration status (χ²=203.0), and year (all *p* < 0.05). The highest need was observed in 2023.

**Fig. 3:**
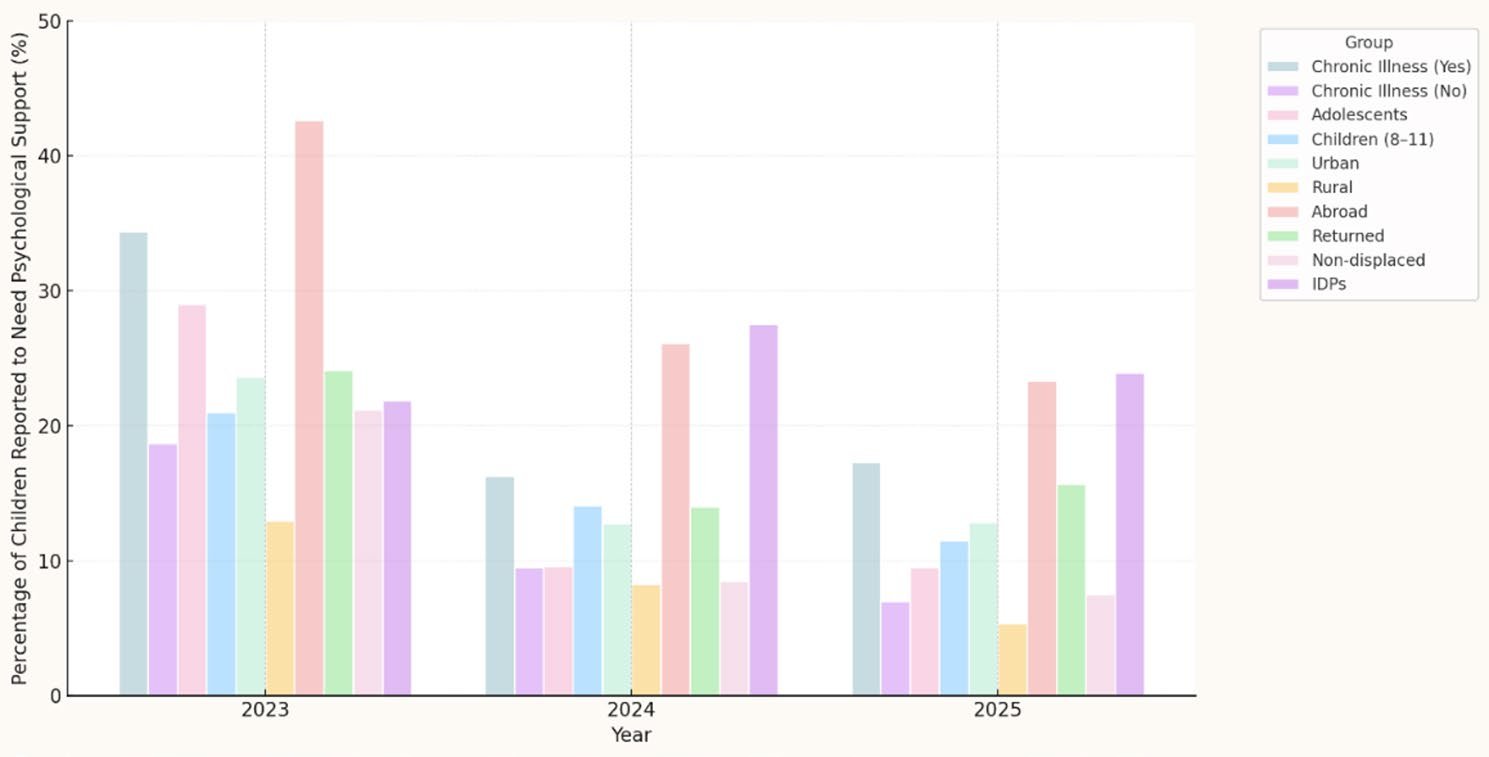
Trends in the reported need for psychological support among school-age children across key subgroups (2023–2025). *The highest levels were observed in 2023, particularly among children with chronic illnesses, adolescents, internally displaced families (IDPs), and families who had moved abroad. A marked decline was noted across most groups in subsequent years*

Figure 3 illustrates temporal patterns in perceived need.

**Fig. 4:**
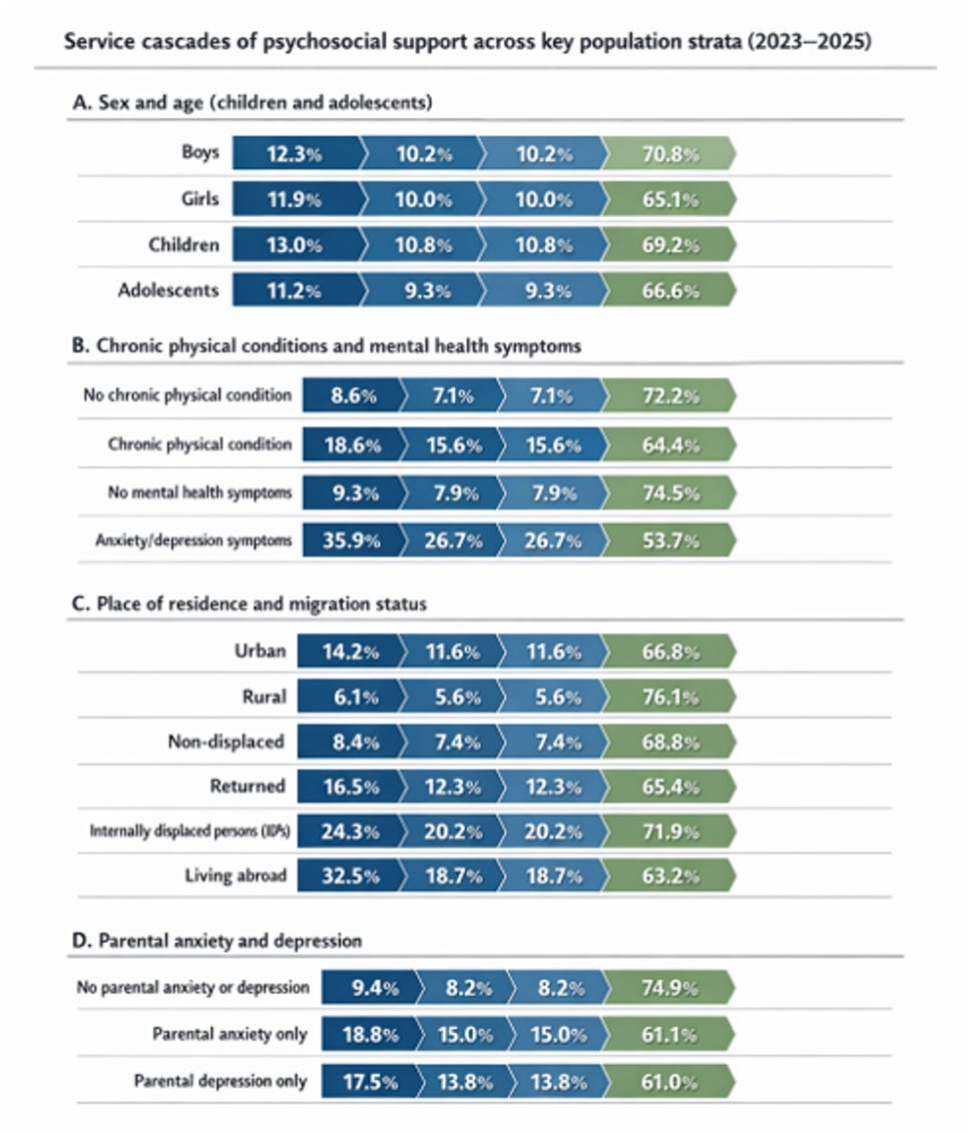
Service cascades of psychosocial support by population subgroup (2023–2025) . Weighted cascade estimates are presented in Supplementary Appendix D, Table D5-9

As shown in Fig. 4, approximately one third of parents acknowledged a need for psychological support, despite higher prevalence of elevated symptoms. This reflects a symptom–need interpretation gap. Supporting cross-tabulations are provided in Appendix D (Tables D3–D5).

Only 9.9% of parents sought help from mental health professionals across 2022–2025. An additional 0.2% consulted somatic specialists such as neurologists or family physicians. Help-seeking increased from 6.9% in 2022 to 10.2% in 2025 (*p* < 0.01). No differences were observed by sex or BMI. However, significant differences were found by migration status, residence, education, chronic illness, and child age (all *p* < 0.05).

Perceived helpfulness among those who sought support was 68.1%. It did not vary by sex, age, year, residence, or migration status (*p* > 0.1). Lower perceived helpfulness was observed among children with chronic conditions and elevated symptoms.

Open-ended responses from 61 parents who did not seek psychological support despite reporting trauma exposure were grouped into six descriptive categories (Appendix D). Common themes included reliance on self-help or prior training (18.6%), intention to seek help in the future (11.5%), limited access to in-person consultations and discomfort with online formats (11.5%), and child refusal (6.5%). One third consulted non-psychologist clinicians instead.

Among respondents, 78.7% were aware of free online or telephone consultations, but only 7.0% utilized them.

### School-Based interventions

Between 2022 and 2025, schools implemented short-format psychological support activities (“psychological minutes”). A related population-based study [19] showed that regular participation in these activities reduced the odds of depressive symptoms by 28.2% (OR = 0.78; 95% CI: 0.61–0.99) and anxiety symptoms by 26.6% (OR = 0.79; 95% CI: 0.57–1.08).

Awareness of these interventions decreased by 29.6% in 2023/2024 compared with the previous period, with the lowest awareness among IDPs families.

### Social support

Informal (horizontal) support was generally moderate to high. Across 2022–2025, 6.9 ± 0.3% rated it as low, 10.1 ± 0.4% as below average, 26.7 ± 0.5% as average, 31.9 ± 0.5% as above average, and 24.4 ± 0.5% as high (mean 3.6 ± 1.2). IDPs and families of children with chronic illnesses reported significantly lower informal support (*p* < 0.001). Rural respondents reported slightly lower support than urban respondents (3.5 vs. 3.6; *p* = 0.006). The average score for informal support from family, friends, and volunteers was 3.6 (SD = 1.2), corresponding to a level between “moderate” and “above average” (Appendix D, Table D10). This rating remained stable across years, age groups, and sexes, suggesting a consistent presence of horizontal support throughout the wartime period.

Institutional (vertical) support was rated considerably lower: 45.8 ± 0.7% rated it as low, and only 3.9 ± 0.3% rated it as high, with a mean of 2.0 ± 1.1. Lowest ratings were seen among IDPs and families of children with chronic illnesses. The average score for institutional support was 2.0 (SD = 1.1), corresponding to a level “below average.”

**Fig. 5:**
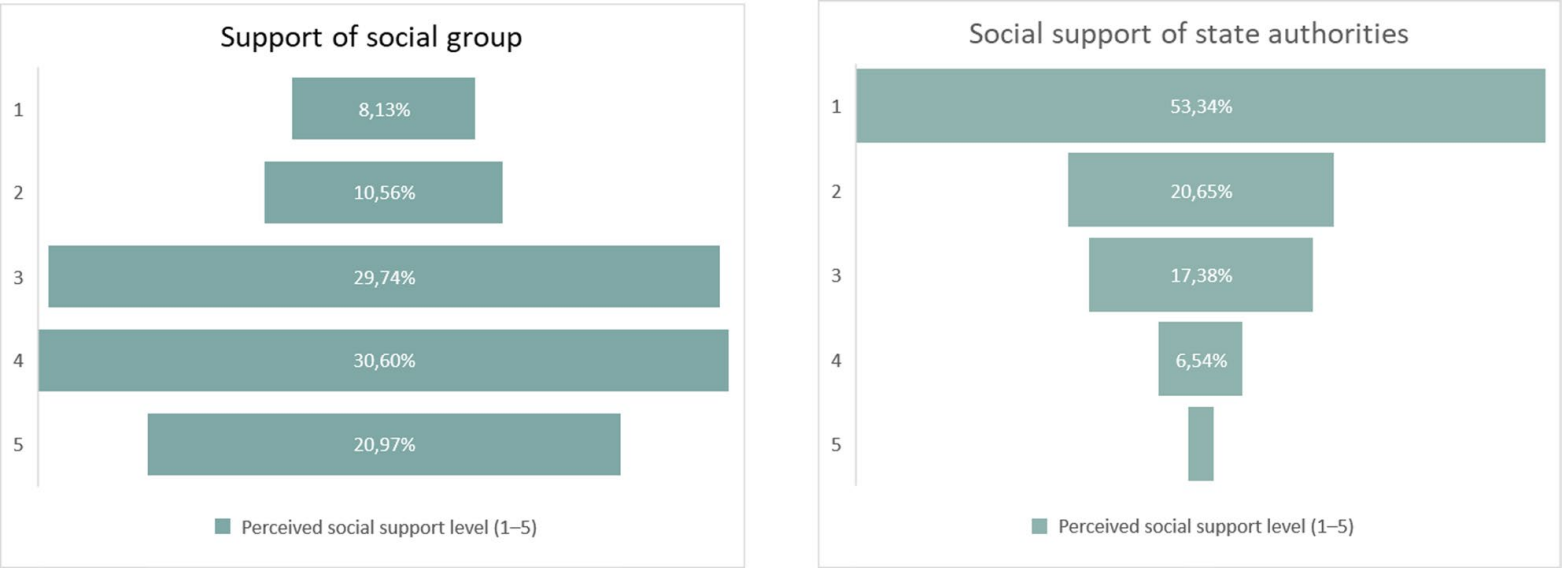
Distribution of perceived social support levels from informal (left) and institutional (right) sources, 2024–2025

Figure 5 contrasts informal and institutional support levels in 2024–2025

### Integrated assessment of psychosocial support for children and adolescents during wartime

Between 2022 and 2025, descriptive analyses indicated a steady increase in parental help-seeking for psychological support for children and adolescents. In adjusted logistic regression models, survey year was specified as a categorical variable with 2025 as the reference category. In the adjusted logistic regression model, survey year was significantly associated with help-seeking behavior (*p* < 0.001), with lower odds observed in 2022 (OR = 0.30; 95% CI: 0.15–0.62) and 2023 (OR = 0.47; 95% CI: 0.36–0.61) compared with the 2025 reference year, while no significant difference was found for 2024 (OR = 0.97; 95% CI: 0.78–1.20) (Table 3).

**Table 3:** Probability of receiving consultations from mental health professionals and the perceived helpfulness of psychological assistance for school-age children

Predictor	Help-seeking OR (95% CI)	*p*	Perceived helpfulness OR (95% CI)	*p*
Year (ref = 2025)				
2022	0.30 (0.15–0.62)	0.001	—	—
2023	0.47 (0.36–0.61)	< 0.001	0.77 (0.45–1.32)	0.336
2024	0.97 (0.78–1.20)	0.764	0.91 (0.58–1.42)	0.678
Migration status (ref = IDP)				
Non-displaced	0.34 (0.27–0.43)	< 0.001	0.90 (0.56–1.45)	0.676
Returned	0.48 (0.37–0.62)	< 0.001	0.86 (0.51–1.46)	0.582
Abroad	0.94 (0.59–1.51)	0.805	0.83 (0.32–2.14)	0.701
Region	1.05 (0.96–1.14)	0.338	1.00 (0.83–1.20)	0.962
Place (rural vs. urban)	0.56 (0.44–0.73)	< 0.001	1.50 (0.83–2.70)	0.179
Age group (adolescents vs. 8–11 yrs)	0.77 (0.64–0.92)	0.004	1.08 (0.74–1.58)	0.689
Sex (female vs. male)	0.98 (0.83–1.15)	0.777	0.70 (0.49–1.00)	0.049
BMI	1.01 (0.98–1.03)	0.722	0.97 (0.92–1.02)	0.279
Chronic disease	1.85 (1.56–2.19)	< 0.001	0.79 (0.55–1.13)	0.200
Education (ref = higher)				
Vocational education	0.92 (0.68–1.24)	0.569	0.61 (0.29–1.26)	0.182
General secondary education	0.81 (0.58–1.13)	0.209	0.56 (0.25–1.25)	0.155
Perceived social support	1.00 (0.93–1.07)	0.934	1.31 (1.12–1.53)	0.001
Child anxiety/depression	3.31 (2.69–4.09)	< 0.001	0.55 (0.37–0.81)	0.002
Parental anxiety	1.24 (1.01–1.53)	0.046	0.74 (0.49–1.14)	0.169
Parental depression	1.12 (0.91–1.37)	0.274	0.88 (0.57–1.34)	0.543

Perceived helpfulness was assessed from 2023 onward; therefore, estimates for 2022 are not available for this outcome

Model fit was acceptable for both logistic regression models. For help-seeking, the model demonstrated moderate explanatory power (Nagelkerke R² = 0.136) and good calibration (Hosmer–Lemeshow *p* = 0.201), with a significant overall likelihood ratio test (χ² = 453.7, *p* < 0.001). Model fit indices further supported adequacy of the specification (AIC = 4971.2; BIC = 4978.2).

For perceived helpfulness, explanatory power was comparable (Nagelkerke R² = 0.122), calibration was adequate (Hosmer–Lemeshow *p* = 0.255), and the overall model fit was statistically significant (χ² = 60.4, *p* < 0.001). nformation criteria indicated a good balance between model fit and parsimony (AIC = 878.7; BIC = 883.3).

Model discrimination was assessed using receiver operating characteristic (ROC) curves. Discrimination was good for the help-seeking model (AUC = 0.732; 95% CI: 0.711–0.752) and moderate for the perceived helpfulness model (AUC = 0.684; 95% CI: 0.642–0.726), indicating adequate ability of both models to distinguish between outcomes.

Migration status was also significant overall (*p* < 0.001): compared with internally displaced families, lower odds of help-seeking were observed in two non-IDP migration groups (OR = 0.34; 95% CI: 0.27–0.43 and OR = 0.48; 95% CI: 0.37–0.62), whereas one subgroup did not differ significantly from IDPs.

Living in a rural area was associated with lower odds of help-seeking compared with urban residence (OR = 0.56; 95% CI: 0.44–0.73).

Parents of adolescents were less likely to seek support than parents of younger children (OR = 0.77; 95% CI: 0.64–0.92), while child sex and BMI were not associated with help-seeking. Children with chronic conditions (OR = 1.85; 95% CI: 1.56–2.19) and those with elevated anxiety or depressive symptoms (OR = 3.31; 95% CI: 2.69–4.09) had substantially higher odds of referral.

Parental anxiety was independently associated with increased help-seeking (OR = 1.24; 95% CI: 1.00–1.53), whereas parental depressive symptoms and perceived social support were not significant predictors.

To assess the robustness of observed associations to differences in sample composition across survey waves, we conducted additional models excluding the 2022 wave. The direction and magnitude of key associations remained largely unchanged (Supplementary Tables S2–S4).

In the adjusted logistic regression model, perceived helpfulness of psychological support was not associated with survey year (overall *p* = 0.627), with no significant differences observed for 2022 (OR = 0.77; 95% CI: 0.45–1.32) or 2023 (OR = 0.91; 95% CI: 0.58–1.42) compared with the 2025 reference year.

Migration status was not associated with perceived helpfulness overall (*p* = 0.953), and no migration subgroup differed significantly from internally displaced families.

Perceived helpfulness was also not associated with region, place of residence, age group, body mass index, chronic health conditions, or parental education.

Female sex was associated with lower odds of perceiving support as helpful (OR = 0.70; 95% CI: 0.49–1.00).

Higher perceived social support was independently associated with greater perceived helpfulness (OR = 1.31; 95% CI: 1.12–1.53). In contrast, families of children with elevated anxiety or depressive symptoms were significantly less likely to report that received support was helpful (OR = 0.55; 95% CI: 0.37–0.81), while parental anxiety and depressive symptoms were not significant predictors.

The regional indicator was not independently associated with either help-seeking (OR = 1.05; 95% CI: 0.96–1.14; *p* = 0.338) or perceived helpfulness (OR = 1.00; 95% CI: 0.83–1.20; *p* = 0.962).

Interaction terms between migration status and place of residence, as well as between child mental health symptoms and perceived social support, were tested in models of perceived helpfulness. Neither interaction was statistically significant (*p* = 0.598 and *p* = 0.179, respectively), and inclusion of interaction terms did not improve model fit. The main effect of perceived social support remained robust, while child symptom status was no longer independently associated with perceived helpfulness in the interaction model.

Interaction terms between migration status and place of residence, as well as between child mental health symptoms and perceived social support, were tested in models of help-seeking. Neither interaction was statistically significant (*p* = 0.784 and *p* = 0.420, respectively), and inclusion of interaction terms did not improve model fit. The association between child anxiety/depressive symptoms and help-seeking remained strong and robust (OR = 4.11; 95% CI: 2.35–7.18).

Examination of predicted probabilities (Table 3) confirmed that the presence of anxiety or depressive symptoms substantially increased the likelihood of help-seeking, whereas higher perceived social support was associated with a higher probability of reporting helpful support. o address potential sampling imbalances, multivariable models were additionally tested with post-stratification weights reflecting sex, educational stage, and urban–rural distribution. Weighted analyses yielded comparable results, confirming the stability of core associations between child mental health symptoms, help-seeking patterns, and perceived helpfulness (see Appendix F). These findings reinforce the interpretive validity of the unweighted models presented below.

### Model for improving psychological support for children and adolescents in Ukraine

Based on the empirical findings of the present study, including identified gaps between mental health symptoms, parent-reported need for psychological support, service utilization, and perceived helpfulness, we propose a model for improving psychosocial support for children and adolescents in Ukraine. The model is explicitly grounded in observed service cascade patterns, subgroup disparities, and multivariable analyses, rather than normative assumptions about service provision.

To inform the structure of the proposed model, we conducted stratified descriptive analyses examining how perceived helpfulness of psychological support varied by levels of social support across key subgroups, including migration status, place of residence, chronic physical illness, and child mental health symptom status. These analyses were complemented by cascade visualizations and adjusted regression models, which together revealed systematic losses at different stages of the support pathway.

As shown in Appendix E, higher perceived social support was consistently associated with greater perceived helpfulness of psychological support; however, the magnitude and statistical significance of this association varied substantially across contexts. The strongest associations were observed among families of children with chronic physical conditions and among urban residents, whereas effects were attenuated or non-significant in rural settings and among families residing abroad. These context-specific patterns indicate that social support can enhance perceived effectiveness but is insufficient to compensate for structural and clinical gaps in high-burden contexts. These findings directly informed the differentiated structure of the proposed model, underscoring the need for tailored rather than uniform psychosocial support pathways.

**Fig. 6:**
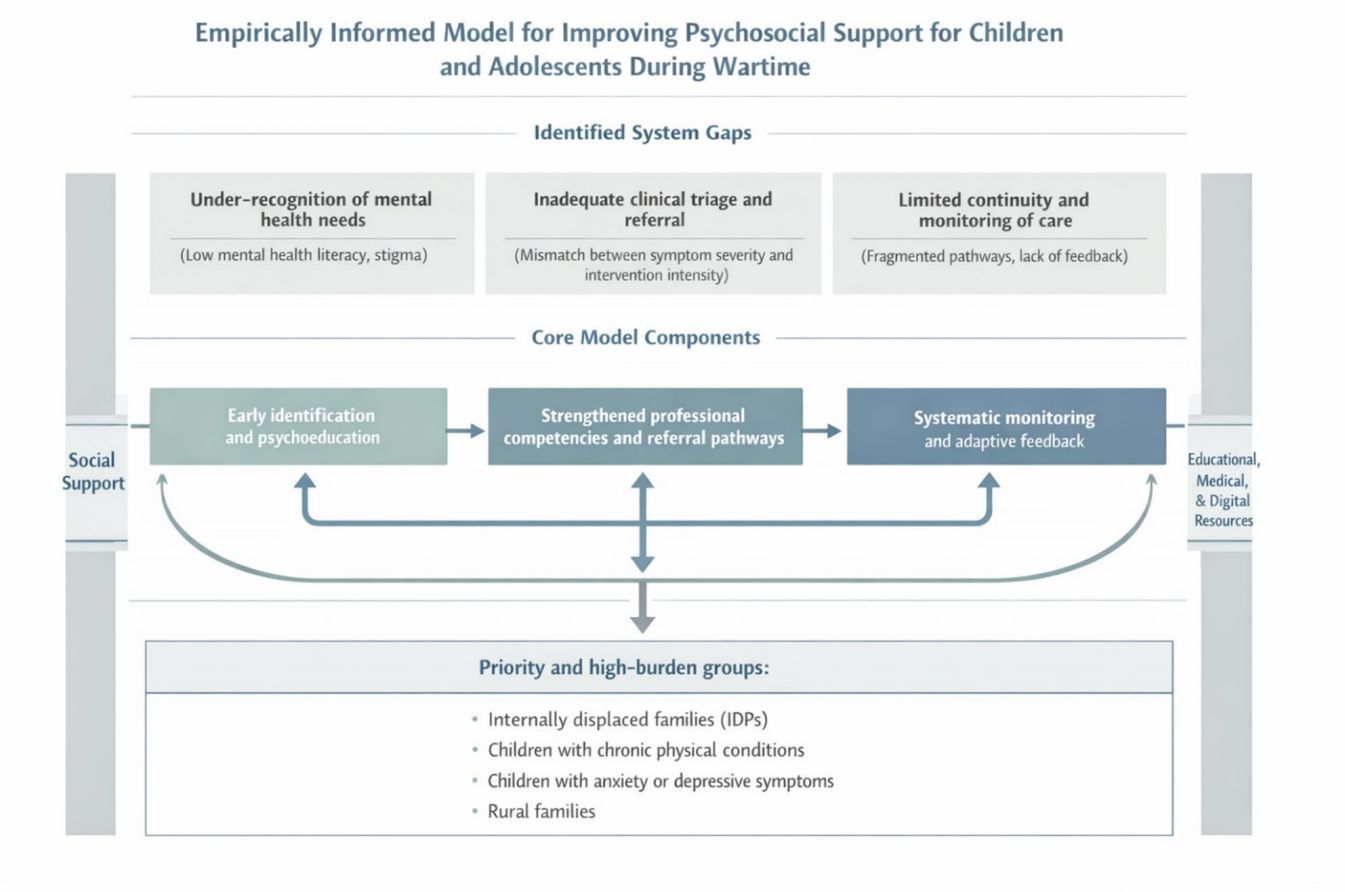
An empirically informed model addressing gaps in psychosocial support for children and adolescents in wartime Ukraine

The model presented in Fig. 6 integrates educational, medical, social, and digital components and is designed to address three empirically identified system-level gaps: (1) under-recognition of mental health needs, (2) inadequate clinical triage and referral, and (3) limited continuity and monitoring of care.

First, the model emphasizes early identification of need through psychoeducation and low-threshold self-help strategies. This component is informed by the observed discrepancy between the prevalence of anxiety and depressive symptoms and the substantially lower proportion of parents who reported a need for psychological support, particularly in rural settings. Although many parents accurately recognized observable symptoms in standardized screening tools, the majority did not interpret these signs as requiring professional intervention. This disconnect, conceptualized as the Symptom–Need Interpretation Gap, represents a critical early barrier in the psychosocial support cascade. Educational programs, workshops, and accessible digital resources aimed at improving mental health literacy may help caregivers better recognize emotional and behavioral symptoms as warranting professional attention. Strategies that reduce apprehension during initial contact with psychological services are especially important in contexts where stigma, normalization of distress, or reliance on informal coping remain prevalent. Targeted outreach is particularly relevant for families returning from displacement and families of military personnel, who experience elevated psychosocial burden.

Second, the model highlights the need to strengthen competencies among helping professionals and improve clinical triage. This direction is directly informed by findings showing that children with elevated anxiety or depressive symptoms were substantially more likely to access services but significantly less likely to receive support perceived as helpful. These patterns suggest a mismatch between symptom severity and the intensity or appropriateness of interventions received. Strengthening training for psychologists, social workers, and primary care providers, alongside clearer referral pathways to specialized mental health care, may improve alignment between identified needs and delivered services. Improving professional competencies may also enhance service quality while mitigating provider overload and burnout.

Third, the model incorporates systematic monitoring and feedback mechanisms, informed by repeated cross-sectional survey waves conducted between 2022 and 2025 that demonstrated temporal variability in mental health indicators and service utilization. Regular monitoring would enable timely identification of emerging needs, evaluation of service availability and perceived quality, and context-responsive service adjustments to changing contextual conditions during prolonged crisis. User-reported feedback is a central element of this component, ensuring that service adjustments reflect lived experiences rather than administrative metrics alone.

Together, these three strategic directions constitute an empirically grounded framework for strengthening the effectiveness, equity, and continuity of psychosocial support systems for children and adolescents during wartime. The model emphasizes coordinated care pathways that facilitate timely progression from low-threshold psychosocial support to specialized mental health services for children with probable clinical symptoms, particularly among internally displaced families and children with chronic health conditions.

## Discussion

Across many countries, prolonged crises, including the COVID-19 pandemic, economic instability, and armed conflicts, have contributed to rising levels of psychological distress and increased demand for MHPSS [28]. International surveys consistently show elevated rates of anxiety, depressive symptoms, sleep disruption, and emotional exhaustion among adults and children, accompanied by growing reliance on informal coping strategies and community-based support networks [12, 29, 30]. These global trends highlight a widening gap between population-level mental health needs and the availability of accessible, acceptable support services.

Against this backdrop, Ukraine shows patterns that are both consistent with and intensified by wartime conditions. Sociological data indicate that self-initiated psychological consultations increased from 7% in 2022 to 17% in 2024, reflecting greater awareness of mental health needs and persistent exposure to chronic stressors [31].

Our findings mirror this general rise: among Ukrainian parents of school-aged children, the rate of help-seeking increased from 6.9% to 10.2% across the study period, with the highest rates observed among internally displaced families and those who had relocated abroad. Notably, help-seeking behavior was not associated with the child’s gender. Parents residing in rural areas were twice as unlikely to seek psychological support compared to those in urban settings. This may reflect greater engagement in urban psychological support groups and related activities an important consideration for future planning [30].

Despite the upward trend, the overall rate of psychological consultations in Ukraine remains lower than in other countries. For example, according to CDC data, approximately 7% of children aged 3–17 in the United States received mental health services in 2022–2023, while rates in the UK, Canada, and Australia were 15%, 10%, and 13%, respectively [32–34].

In our study, 9.9% of Ukrainian parents sought psychological support for their children between 2022 and 2025, a figure comparable to the 7.9% reported in the 2022–2023 National Survey of Children’s Health in the U.S [32]. Among children with anxiety and depressive symptoms, 21.7% of parents sought professional help, compared to 7.3% in the group without such symptoms. In contrast, in the UK, 76.2–76.5% of parents of children with mental health concerns sought advice or consultation from mental health professionals during the same period [33].

All parents in our sample who sought psychological support for their children reported receiving it. Among those who believed their child needed help, nearly 50% contacted mental health professionals. A small proportion (0.2%) consulted not only psychologists but also family doctors, neurologists, and other somatic specialists. According to the National Institutes of Health (US), approximately 82.6% of adolescents in need of mental health care in the U.S. receive it through psychological services [32]. These comparisons suggest that psychological support in Ukraine is generally accessible, though certain aspects require improvement.

Perceived helpfulness of psychological support was reported by 68.1% of parents overall, regardless of the child’s gender, age, migration status, or parental education. However, among parents of children screening positive for anxiety and depressive symptoms, perceived helpfulness was substantially lower 1.4 times lower than among those whose children had other psychological concerns (53.9% vs. 74.6%).

These findings suggest that while psychosocial support may be relatively effective for children with mild or situational distress, substantial unmet needs persist among children with probable clinical symptomatology the group most in need of care. Although our study did not assess outcomes of specific therapeutic interventions, approximately 75% of patients report psychotherapy as effective [32].

The observed discrepancy underscores the importance of stepped-care approaches and clearer referral pathways, ensuring timely transition from low-threshold psychosocial support to specialized mental health services for children with persistent or severe symptoms [1]. This also highlights the need for training helping professionals to recognize borderline and clinical presentations and initiate appropriate referrals. Recent initiatives in Ukraine aimed at strengthening collaboration between mental health specialists and other helping professionals represent important progress [11–13, 35], however, our findings indicate that further development of intersectoral cooperation remains essential.

Importantly, the observed temporal changes in the prevalence of elevated anxiety and depressive symptoms should not be interpreted as evidence of intervention effectiveness or population-level adaptation in our study. The present study did not assess treatment exposure or therapeutic outcomes, and help-seeking behavior was analyzed separately from symptom prevalence. Given the repeated cross-sectional design and evolving contextual conditions, changes in prevalence may reflect multiple alternative processes, including sampling differences across survey waves, shifts in parental awareness and reporting thresholds, changes in exposure to acute stressors, or measurement-related artifacts.

The lack of regional differences in help-seeking and perceived helpfulness should be interpreted in light of the nationwide nature of war exposure in Ukraine. While regional variation was evident for anxiety and depressive symptoms, reflecting differences in cumulative stress and local context, access to and perceived effectiveness of psychosocial support did not vary by region after adjustment. This suggests that, suggests that during prolonged conflict, regional location may be less informative for service-related outcomes than individual symptom burden, migration experience, and social support.

Parents cited a range of perceived barriers to psychological support, including preference for self-help, lack of offline options, distrust toward mental health services, or the child’s refusal to participate. One-third consulted other professionals such as neurologists or family doctors instead. These qualitative patterns were consistent with prior surveys from wartime Ukraine, in which families most often reported limited availability of services (27.8%), long waits (19.4%), stigma (14.6%), and financial constraints (11.2%) [12]. Notably, despite 78.7% of parents being aware of free hotline or online services, only 7.0% used them, highlighting a gap between awareness and engagement. Such findings underscore the importance of trust-building, flexibility in service formats, and culturally responsive, low-threshold mental health support in crisis settings.

The widespread implementation of psychological tools in schools, known as “psychological minutes”, was initiated by the Ministry of Education and Science of Ukraine in 2022–2023 to stabilize children’s emotional states during wartime [18]. Our previous research showed that teachers’ use of various well-being strategies helped prevent anxiety and depressive symptoms. However, parental awareness of these interventions declined by 29.6% in 2024 compared to 2023 [19]. Given the school’s central role in shaping children’s mental health, and the scale of these interventions, regular monitoring and population-level evaluation are needed.

Digital mental health services, including AI-based chatbots, are rapidly developing in Ukraine [15, 16]. However, legal frameworks in this area require refinement and clearer regulation. Public awareness of safety protocols for online consultations must be improved, alongside efforts to reduce stigma around digital formats of psychological support.

Importantly, mental health outcomes are shaped not only by professional services but also by broader societal dynamics. Society can be both a source of stress and a remedy for it and community support is a critical factor in addressing psychological challenges during emergencies [1, 28]. Research has shown that social support functions as a unifying concept across all societal levels, strengthening individual and family resilience and activating community resources that promote adaptation and well-being [30, 31, 36, 37]. Thus, perceived social support serves as both an indicator of societal cohesion and a measure of psychosocial intervention perceived helpfulness.

Following the full-scale invasion, Ukrainian society demonstrated remarkable solidarity, reflected in mutual support, patriotism, and trust, both offline and online [11, 12, 15, 16, 36]. In our survey, half of respondents reported high or above-average levels of social support from their communities, while only one in fourteen reported low support. The highest levels were observed among respondents who remained abroad throughout the study, although this rate declined significantly between 2023 and 2025. Internally displaced families also reported a decrease in perceived support. Nearly one in ten families with school-aged children reported below-average support, signaling that a substantial segment of the population lacks access to essential resources and requires further attention.

State support was rated at an average of 2 out of 5 in 2024–2025, with nearly half of respondents giving low scores and only 3.9% reporting high levels. This highlights the dominance of close interpersonal connections over institutional support during crisis. Respondents tended to rely on accessible, emotionally resonant relationships rather than formal structures. The decline in institutional ratings reflects limited trust in state mechanisms, while the rise in 2025 compared to 2024 suggests emerging top-down improvements.

Logistic regression models confirmed inequalities in access to psychological support and social resources, underscoring the complexity of help‑seeking behavior and emotional well‑being during crisis. The strongest predictor of help‑seeking was the presence of anxiety and depressive symptoms in children, which significantly increased the likelihood of parental engagement with psychological services. Parental anxiety also emerged as an independent predictor of help-seeking, suggesting that caregivers’ emotional state influences their recognition of need and readiness to act. In contrast, parental depressive symptoms and perceived social support were not associated with help-seeking.

Our findings that higher levels of social support were associated with greater help‑seeking and perceived helpfulness are consistent with previous studies showing that supportive environments facilitate access to care and improve satisfaction with services. At the same time, the association between social support and perceived helpfulness was not uniform across subgroups, indicating that contextual and structural conditions may moderate how families experience and evaluate psychosocial services. These findings indicate that social support alone is insufficient as a universal mechanism and must be embedded within broader, context-sensitive systems of care.

Based on these results, we developed a model for improving psychological support for children and adolescents in Ukraine. The model integrates educational, medical, social, and digital components and is designed to identify needs, reduce access barriers, and provide targeted support to vulnerable groups, particularly children in crisis. It offers a framework for advancing intersectoral collaboration in mental health care.

A key strength of this study is its focus on direct feedback from service users—parents of school-aged children. This subjective perspective complements existing monitoring systems and is particularly relevant in emergency settings, where formal outcome measures may fail to capture the dynamic and evolving nature of children’s psychosocial symptoms under ongoing trauma. Consistent with recommendations of the Inter-Agency Standing Committee of the United Nations [28], the study prioritizes feasibility and contextual relevance over narrowly defined clinical endpoints.

The use of a service cascade framework integrating perceived need, help-seeking, access to services, and parent-reported perceived helpfulness allows identification of where losses occur along the support pathway and which groups face the greatest gaps. While the dichotomous item assessing perceived helpfulness is limited, it reflects the reality that in crisis contexts effectiveness is often defined not solely by symptom reduction, but by the provision of safety, hope, and attentive listening as experienced by families.

By explicitly distinguishing between psychosocial support suitable for mild or situational distress and the needs of children with probable clinical symptomatology, the study strengthens its public health relevance and supports the application of stepped-care approaches and differentiated referral pathways in wartime settings.

Several limitations should be acknowledged. First, the study relied on non-probability sampling, particularly in the early survey waves, and was conducted entirely online, which may limit representativeness. In the context of large-scale displacement and population mobility during wartime, traditional indicators of population structure (e.g., urban–rural distribution) are subject to substantial uncertainty. Body mass index was calculated from parent-reported anthropometric data and may be subject to measurement error.

Second, evaluations of children’s mental health and psychosocial support were based exclusively on parent report. While the RCADS-P-25 primarily captures observable emotional and behavioral symptoms such as avoidance, agitation, or sleep disruption that caregivers are well-positioned to recognize, the absence of multi-informant data limits interpretation of children’s internal states. Additionally, wartime conditions may heighten parental vigilance and protective instincts, influencing sensitivity to children’s emotional cues.

Our findings suggest that many parents did recognize clinically significant symptoms; however, only one-third of them perceived a need for professional support. This notable discrepancy between symptom recognition and help-seeking behavior conceptualized in this study as the Symptom–Need Interpretation Gap (SNIG) may reflect normalization of distress, stigma, or limited mental health literacy. While parent report has inherent limitations, it remains a valuable and often the only feasible source of insight into children’s mental health in humanitarian settings where clinical assessments are inaccessible.

Third, participation required access to digital communication channels, introducing potential self-selection bias. Although online surveys may limit opportunities for clarification and follow-up, in contemporary Ukraine they represent a widely accepted and culturally appropriate means of communication between schools, parents, and communities. Early survey waves may overrepresent parents who were more digitally connected or motivated to engage during periods of heightened uncertainty.

Fourth, the repeated cross-sectional design and variation in sampling strategies across survey waves limit the interpretation of temporal differences. Observed changes in mental health indicators or support pathways may reflect differences in sample composition, parental awareness, exposure to acute stressors, or measurement-related artifacts rather than population-level trends or intervention effects. To ensure accurate interpretation, all between-year comparisons are framed as descriptive rather than longitudinal.

Moreover, while recruitment from 2023 onward was facilitated through schools, the survey remained anonymous and no school-level identifiers were collected. As such, potential intra-school clustering could not be explicitly modeled. However, schools functioned solely as dissemination channels and were not treated as analytical units. Finally, although regional indicators were included as covariates, region was not independently associated with outcomes after adjustment. Given the widespread impact of war-related stressors across Ukraine, including regular air raid alerts and missile attacks, regional variation in access or perceived helpfulness appears limited and is likely overshadowed by individual- and household-level factors.

Taken together, these findings emphasize that resilience in wartime contexts should be conceptualized as a multidimensional process shaped by psychosocial resources, community support, and system-level responsiveness, rather than by symptom reduction alone. While the present study focused on psychosocial indicators, future research should integrate psychosocial and biological perspectives to better understand vulnerability, help-seeking behavior, and perceived helpfulness of support under prolonged crisis conditions.

## Conclusion

The findings indicate a relatively high level of parent-reported perceived helpfulness of psychosocial support at the population level. However, perceived effectiveness was substantially lower among families with children experiencing anxiety or depressive symptoms - the group most in need of care. This discrepancy underscores critical gaps in the current system of psychosocial support.

The results highlight the need to strengthen training for medical and helping professionals, particularly in the prevention, early recognition, and management of mental health concerns in children and adolescents. Establishing clearer referral pathways is essential to ensure timely transitions from basic psychosocial support to specialized care.

Persistent inequalities in access to psychosocial support remain a priority concern, especially for families living in rural areas and those caring for children with elevated symptom burden. Internally displaced children represent a high-risk group not merely due to their status, but due to disproportionately higher rates of psychological distress, warranting targeted and needs-based interventions.

## Supplementary Information

Supplementary Material 1.

## Data Availability

The datasets used and/or analyzed during the current study are available from the corresponding author upon reasonable request.

## Abbreviations

BMI: Body Mass Index
MHPSS: Mental Health and Psychosocial Support
RCADS-P-25: Revised Child Anxiety and Depression Scale - Parent Version
Non-displaced: Families not displaced due to hostilities
Returned: Families temporarily relocated but returned to previous residence
Abroad: Families displaced due to hostilities and residing outside Ukraine at the time of the survey
IDPs: Internally Displaced Persons (families displaced within Ukraine)
