# Supplementary material for: Psychosocial support for schoolchildren in wartime Ukraine: community-based access and parent-reported perceived helpfulness
Source: Confl Health. 2026 Feb 8;20:25. doi: 10.1186/s13031-026-00762-9 (PMC12983549; doi:10.1186/s13031-026-00762-9)
Supplement: Supplementary file 1 — Supplementary Material 1. [file 13031_2026_762_MOESM1_ESM.docx]

Supplemental materials

**Table of contents**

[**Appendix A. Methodological validity of online parent surveys in wartime Ukraine** 1](#_Toc219585740)

[**Appendix B. Sample Characteristics and Post-Stratification Weights** 2](#_Toc219585741)

[**Appendix C. Reliability and Adaptation of Revised Child Anxiety and Depression Scale – Parent Version (RCADS-P-25) in Ukraine** 4](#_Toc219585742)

[**Appendix D. Descriptive and Weighted Subgroup Analyses** 8](#_Toc219585743)

[**Appendix E. Stratified associations between social support and perceived helpfulness of psychological support** 20](#_Toc219585744)

[**Appendix F. Exploratory logistic regression models: interaction terms and weighted analyses** 21](#_Toc219585745)

# **Appendix A. Methodological validity of online parent surveys in wartime Ukraine**

The choice of an online survey as the primary data collection method in this study reflects both the technological capacity of the Ukrainian population and the practical constraints imposed by wartime conditions. As of 2024, 95% of Ukrainian households have internet access, and 87% of the population owns smartphones (State Statistics Service of Ukraine, 2024). Digital inclusion is especially high among families with school-aged children, who are accustomed to digital school communication through electronic journals, messaging platforms, and government-supported educational tools. These practices became entrenched following the national transition to remote and hybrid learning formats, formalized in Order No. 1115 (Ministry of Education and Science, 2020).

During 2022–2025, this digital infrastructure remained functional even amid displacement and instability. Online parent–teacher communication became the default across both urban and rural schools, including group chats for classrooms, subject-specific discussions, and individual feedback from teachers. In this context, inviting parents to participate in an online survey closely mirrored their regular educational interactions and thus ensured cultural and logistical appropriateness. Importantly, the reach of online surveys extended to internally displaced and repeatedly relocated families, contributing to broad socio-demographic coverage.

Beyond accessibility, the asynchronous survey format addressed key wartime challenges, such as power outages, air-raid alarms, and emotional strain. Parents could complete the questionnaire over multiple sessions, which was explicitly supported in the study design and contributed to realistic participation. While self-selection bias is acknowledged, primarily in relation to parental motivation and digital confidence, it does not undermine the fundamental validity of the method, given the near-universal digital access among the target group.

In sum, the use of online surveys in this study represents a methodologically sound, ethically sensitive, and context-appropriate approach for population-level data collection during protracted conflict. It reflects the lived communication environment of Ukrainian families and offers an ecologically valid snapshot of child well-being as perceived by parents navigating the realities of war.

# **Appendix B. Sample Characteristics and Post-Stratification Weights**

*This appendix presents summary distributions and post-stratification weights applied to adjust for sampling imbalances across place of residence, sex, and educational level (grade band). For each year (2022–2025), official statistics were obtained from the Ministry of Education and Science of Ukraine and used to calculate ratio-based weights for key variables. These weights were applied separately by year in sensitivity analyses.*

**Table B1. Urban and Rural Distributions and Post-Stratification Weights (2022–2025)**

| Year | Place of residence | % in National Population | Sample | Weight |
| --- | --- | --- | --- | --- |
| 2022 | Urban | 0.6942 | 0.8646 | 0.8028 |
| 2022 | Rural | 0.3058 | 0.1354 | 2.2585 |
| 2023 | Urban | 0.6948 | 0.8725 | 0.7965 |
| 2023 | Rural | 0.3052 | 0.1275 | 2.3937 |
| 2024 | Urban | 0.6953 | 0.841 | 0.8267 |
| 2024 | Rural | 0.3047 | 0.159 | 1.9164 |
| 2025 | Urban | 0.6953 | 0.6925 | 1.004 |
| 2025 | Rural | 0.3047 | 0.3075 | 0.991 |

Table B2. Sex Ratios and Post-Stratification Weights (2022–2025)

| Year | Grades | % in National Population | Sample | Weight female | Weight male |
| --- | --- | --- | --- | --- | --- |
| 2022 | Primary education | 0.950 | 0.943 |  |  |
| 2022 | First stage of secondary education | 0.955 | 0.993 |  |  |
| 2022 | Second stage of secondary education | 0.930 | 1.050 |  |  |
| 2022 | Total | 0.945 | 0.979 | 0.965 | 1.036 |
| 2023 | Primary education | 0.952 | 1.070 |  |  |
| 2023 | First stage of secondary education | 0.955 | 0.896 |  |  |
| 2023 | Second stage of secondary education | 0.930 | 0.941 |  |  |
| 2023 | Total | 0.946 | 0.965 | 0.980 | 1.020 |
| 2024 | Primary education | 0.955 | 0.791 |  |  |
| 2024 | First stage of secondary education | 0.952 | 1.044 |  |  |
| 2024 | Second stage of secondary education | 0.934 | 1.213 |  |  |
| 2024 | Total | 0.947 | 0.955 | 0.992 | 1.008 |
| 2025 | Primary education | 0.956 | 0.913 |  |  |
| 2025 | First stage of secondary education | 0.954 | 0.952 |  |  |
| 2025 | Second stage of secondary education | 1.059 | 1.240 |  |  |
| 2025 | Total | 0.968 | 0.961 | 1.007 | 0.993 |

Table B3. Educational Level (Grade Bands) and Post-Stratification Weights (2022–2025)

| Year | Grade Bands | % in National Population | Sample | Weight |
| --- | --- | --- | --- | --- |
| 2022 | Primary education | 0.389 | 0.399 | 0.97494 |
| 2022 | First stage of secondary education | 0.466 | 0.491 | 0.94908 |
| 2022 | Second stage of secondary education | 0.145 | 0.110 | 1.31818 |
| 2022 | Total | 1 | 1 |  |
| 2023 | Primary education | 0.366 | 0.390 | 0.93846 |
| 2023 | First stage of secondary education | 0.485 | 0.502 | 0.96614 |
| 2023 | Second stage of secondary education | 0.150 | 0.108 | 1.38889 |
| 2023 | Total | 1 | 1 |  |
| 2024 | Primary education | 0.347 | 0.360 | 0.96389 |
| 2024 | First stage of secondary education | 0.496 | 0.566 | 0.87633 |
| 2024 | Second stage of secondary education | 0.156 | 0.074 | 2.10811 |
| 2024 | Total | 1 | 1 |  |
| 2025 | Primary education | 0.343 | 0.349 | 0.98281 |
| 2025 | First stage of secondary education | 0.525 | 0.561 | 0.93583 |
| 2025 | Second stage of secondary education | 0.132 | 0.090 | 1.46667 |
| 2025 | Total | 1 | 1 |  |

# **Appendix C. Reliability and Adaptation of Revised Child Anxiety and Depression Scale – Parent Version (RCADS-P-25) in Ukraine**

*This appendix provides detailed information on the translation, cultural adaptation, and psychometric properties of the Ukrainian version of the RCADS-P-25.*

*The instrument was administered using U.S. normative T-scores (≥65), as recommended by the scale developers. A validation study conducted in Ukraine confirmed the appropriateness of these thresholds for the local population. The Ukrainian RCADS-P-25 demonstrated excellent internal consistency (α/ω), high test–retest reliability, and stable psychometric performance across survey years (2022–2025) and age groups.*

*As a parent-reported instrument, the RCADS-P-25 is well suited for population-based screening, particularly in contexts where multi-informant assessment is not feasible.*

***Background.*** In crisis settings, rapid assessment of the mental health of vulnerable populations is essential for timely and targeted psychosocial support. When the number of affected individuals is large, clinical interviews are often infeasible due to limited specialist availability and constraints on in-person contact. Under such conditions, brief standardized instruments suitable for online administration are recommended.

One such instrument is the Revised Child Anxiety and Depression Scale – Parent Version (RCADS-P-25), which is recommended by the United Nations Inter-Agency Standing Committee for assessing child and adolescent mental health in emergency contexts. The RCADS-P-25 comprises 25 items and allows for efficient screening in large-scale population studies.

The Ukrainian adaptation of the RCADS-P-25 was conducted during the COVID-19 pandemic. Its acceptable psychometric properties and good test–retest reliability supported its subsequent use during the wartime period. The suitability of the instrument is further enhanced by its focus on salient emotional, cognitive, and behavioral manifestations (e.g., persistent sadness, excessive worry, sleep and appetite disturbances, avoidance behaviors, fatigue, restlessness, repetitive behaviors) that are observable in everyday functioning. This makes the scale appropriate for population-based research in wartime conditions, even when parents are highly burdened.

***1.1. Translation and Cultural Adaptation Procedure***

The RCADS-P-25 was translated and adapted into Ukrainian following a five-step procedure to ensure linguistic accuracy and cultural relevance:

1. Forward translation by Ukrainian experts fluent in both English and Ukrainian, including a psychologist, a philologist, and one of the study authors.
2. Blind back-translation into English by a bilingual linguist based in the United States.
3. Focus group review with 10 parents and experts to assess clarity, cultural appropriateness, and semantic equivalence.
4. Pilot testing with repeated administration and feedback collection to refine item wording.
5. Expert consensus to finalize the adapted version, ensuring content validity and preservation of the original structure.

Permission for translation and use of RCADS-P-25 was obtained from the original authors, as outlined on the official RCADS website (<https://rcads.ucla.edu/>).

**Adaptation-specific considerations**

During the adaptation process, several wording modifications were introduced to improve clarity and cultural relevance while maintaining conceptual equivalence with the original instrument. The original instruction (“Please put a circle around the word that shows how often each of these things happens for your child”) was replaced with a formulation that was easier for respondents to understand and complete. Parents were also explicitly encouraged to consult with their child when uncertain about an item response.

In contrast to the original clinical framing based on a two-week reference period, the Ukrainian version assessed symptoms over the past month. This modification was considered more appropriate for population-based research conducted under conditions of prolonged instability (pandemic lockdowns and wartime stress), where shorter reference periods may not adequately capture stable behavioral and emotional changes.

Additionally, several items (Q10, Q11, Q15) were expanded to clarify appetite disturbances, vegetative symptoms, and concentration difficulties. Item Q18 was reworded from “My child thinks about death” to “My child talked about death” to reduce ambiguity and improve parental interpretability. All modifications were reviewed and approved by experts to ensure equivalence with the original instrument.

The adapted questionnaire was subsequently administered to the core research sample, enabling further evaluation of its validity and reliability.

***1.2. Confirmatory Factor Analysis***

Given that the original RCADS-P-25 is based on a two-factor structure (Anxiety and Depression subscales), the factorial validity of the Ukrainian version was examined using confirmatory factor analysis (CFA).

CFA was conducted using a categorical data approach to account for the ordinal nature of item responses. Model estimation was performed using the cfa function with parameters appropriate for ordered indicators.

The initial two-factor model demonstrated acceptable fit to the data (CFI = 0.931; TLI = 0.924). Although the χ² statistic was statistically significant (χ² = 1370.9), this was expected given the large sample size, as χ² is known to be highly sensitive to sample size. The RMSEA value was 0.077 (90% CI: 0.073–0.081), indicating acceptable but not optimal fit. The SRMR value (0.092) slightly exceeded the recommended threshold of 0.08, suggesting potential areas for model improvement.

To refine the model, modification indices were examined. Correlated error terms were added between items Q3 and Q9 (MI = 381.9) and between items Q7 and Q22 (MI = 292.7), reflecting shared item content and contextual overlap. The modified model demonstrated substantially improved fit: χ² = 701.5; CFI = 0.972; TLI = 0.969; RMSEA = 0.049 (90% CI: 0.045–0.053); SRMR = 0.075. Both CFI and TLI exceeded the recommended threshold of 0.95, while RMSEA and SRMR indicated good model fit.

Overall, the CFA results confirm that the factorial structure of the Ukrainian RCADS-P-25 corresponds to the original two-factor model. The presence of correlated error terms suggests additional sources of shared variance, which may reflect culturally or contextually specific symptom expressions relevant in crisis settings. These findings support the structural validity of the instrument and provide a robust basis for subsequent reliability estimation using McDonald’s omega.

***1.3. Reliability coefficients***

This appendix presents reliability coefficients (see Table 1) for the Ukrainian adaptation of RCADS-P-25 across the war period (2022–2025). Reliability was assessed separately for anxiety and depression subscales, and stratified by age group: all participants, children (8–11), and adolescents (12–18).

Methods.

- McDonald’s omega (ω) and SEM-based Cronbach’s alpha were calculated using the *semTools* package in R.
- Ordinal alpha was computed using the *psych* package. In some years, ordinal alpha values were unavailable due to sample-specific constraints.
- All reliability coefficients were computed separately for anxiety and depression subscales.
- Sample sizes and status indicators are provided for transparency.

Results.

- Across all years and groups, ω ranged from 0.77 to 0.83 for anxiety and 0.74 to 0.87 for depression.
- SEM-based α values remained stable, ranging from 0.74 to 0.85.
- Ordinal α values were consistently high (0.92–0.94) when available.
- Aggregated data for 2022–2025 (war period) showed ω = 0.79 (Anxiety), 0.81 (Depression); α SEM = 0.80 (Anxiety), 0.81 (Depression); ordinal α = 0.93 (Anxiety), 0.93 (Depression).
- ICC values for test–retest reliability ranged from 0.92 to 0.96.

In our study, T-scores were derived from the original U.S. normative data, as recommended by the authors of RCADS. At the same time, we conducted a Ukrainian validation study (Yelizarova et al., 2024^[[1]](#footnote-1)^), which confirmed that the use of these thresholds is appropriate in the Ukrainian population. Reliability patterns were consistent across study years, with no evidence of systematic degradation during the war period. The validation demonstrated high internal consistency (α/ω) and test–retest reliability, supporting the applicability of the U.S.-based norms in our context.

Table C1. Internal consistency of RCADS-P-25 by year and age band

| Year | Age band | Scale | Omega | Alpha SEM | Alpha Ordinal | N | Status |
| --- | --- | --- | --- | --- | --- | --- | --- |
| 2022 | All | Anxiety | 0.80 | 0.80 | 0.93 | 1108 | OK |
| 2022 | All | Depression | 0.87 | 0.84 | 0.93 | 1108 | OK |
| 2022 | Children | Anxiety | 0.80 | 0.80 | 0.93 | 437 | OK |
| 2022 | Children | Depression | 0.86 | 0.83 | 0.93 | 437 | OK |
| 2022 | Adolescents | Anxiety | 0.81 | 0.82 | 0.94 | 671 | OK |
| 2022 | Adolescents | Depression | 0.87 | 0.85 | 0.94 | 671 | OK |
| 2023 | All | Anxiety | 0.77 | 0.78 | 0.93 | 1544 | OK |
| 2023 | All | Depression | 0.85 | 0.83 | 0.93 | 1544 | OK |
| 2023 | Children | Anxiety | 0.77 | 0.77 | <NA> | 605 | OK |
| 2023 | Children | Depression | 0.81 | 0.80 | <NA> | 605 | OK |
| 2023 | Adolescents | Anxiety | 0.79 | 0.80 | 0.94 | 939 | OK |
| 2023 | Adolescents | Depression | 0.86 | 0.85 | 0.94 | 939 | OK |
| 2024 | All | Anxiety | 0.80 | 0.81 | 0.93 | 1114 | OK |
| 2024 | All | Depression | 0.79 | 0.79 | 0.93 | 1114 | OK |
| 2024 | Children | Anxiety | 0.78 | 0.78 | <NA> | 373 | OK |
| 2024 | Children | Depression | 0.78 | 0.74 | <NA> | 373 | OK |
| 2024 | Adolescents | Anxiety | 0.83 | 0.83 | 0.94 | 741 | OK |
| 2024 | Adolescents | Depression | 0.79 | 0.80 | 0.94 | 741 | OK |
| 2025 | All | Anxiety | 0.78 | 0.80 | 0.93 | 4787 | OK |
| 2025 | All | Depression | 0.77 | 0.78 | 0.93 | 4787 | OK |
| 2025 | Children | Anxiety | 0.78 | 0.80 | 0.93 | 1663 | OK |
| 2025 | Children | Depression | 0.74 | 0.75 | 0.93 | 1663 | OK |
| 2025 | Adolescents | Anxiety | 0.80 | 0.81 | 0.94 | 3124 | OK |
| 2025 | Adolescents | Depression | 0.79 | 0.79 | 0.94 | 3124 | OK |
| 2022-2025 | All | Anxiety | 0.79 | 0.80 | 0.93 | 8553 | OK |
| 2022-2025 | All | Depression | 0.81 | 0.81 | 0.93 | 8553 | OK |
| 2022-2025 | Children | Anxiety | 0.78 | 0.80 | 0.92 | 3078 | OK |
| 2022-2025 | Children | Depression | 0.79 | 0.78 | 0.92 | 3078 | OK |
| 2022-2025 | Adolescents | Anxiety | 0.80 | 0.81 | 0.94 | 5475 | OK |
| 2022-2025 | Adolescents | Depression | 0.82 | 0.82 | 0.94 | 5475 | OK |

*Note: Ordinal Cronbach’s alpha based on polychoric correlations could not be estimated in some year-by-age subgroups due to instability of polychoric correlation matrices in smaller or distributionally constrained samples. Model-based reliability coefficients were available for all subgroups.*

***Conclusion.*** The RCADS-P-25 demonstrates high internal consistency and temporal stability across years and age groups. The consistent reliability metrics support its use in Ukrainian population studies during wartime

# **Appendix D. Descriptive and Weighted Subgroup Analyses**

*This appendix presents a detailed overview of children's mental health, help-seeking behavior, and perceived helpfulness of psychosocial support in wartime Ukraine, based on a nationwide parent survey conducted between 2022 and 2025. Where applicable, subgroup-specific proportions are weighted using post-stratification weights for sex, place of residence, and educational stage (see Appendix B for details). These tables supplement multivariable regression models by highlighting descriptive gradients across key sociodemographic and vulnerability dimensions.*

Cascade of Psychosocial Support: From Symptom Identification to Perceived Helpfulness (2022–2025)

The following tables illustrate a four-stage service cascade, reflecting the progression from mental health symptoms to received psychosocial support:

Elevated anxiety/depression symptoms (screening stage)

→ Table D1 reports the prevalence of anxiety and depressive symptoms by sex and sociodemographic characteristics. During 2022–2025, 12.8% of children screened positive. Rates were highest among children with chronic illness and those from displaced families.

Perceived need for psychological support (need recognition stage)

→ Captured in the main Results section, 12.0% of parents reported that their child needed psychological support—substantially fewer than those screening positive.

Help-seeking behavior (contact stage)

→ Table D3 shows that only 9.9% of respondents contacted a psychologist. Help-seeking was more common among displaced families and parents of younger children.

Perceived helpfulness of received support (outcome perception stage)

→ Table D4 shows that among those who accessed care, 68.1% found it helpful. However, this dropped to 53.9% for parents of children with elevated symptoms.

These cascade stages are further examined through weighted subgroup analyses in Tables D5–D8.

Table Overview

D1–D4: Core service cascade stages

D5–D8: Weighted subgroup comparisons (demographics, health, migration, parent mental health)

D9: Thematic analysis of open-text barriers

D10: Distribution of perceived social support

Table D1 presents the distribution of parent‑reported anxiety and depressive symptoms among school‑age children across survey waves and key sociodemographic subgroups. Overall, 12.8% of children screened positive for anxiety and depressive symptoms during the study period. The proportion was highest in 2022 (24.7%) and decreased to 9.4% in 2025 (χ² = 164.4; p < 0.001).

Elevated symptom prevalence was consistently observed among children with chronic illnesses (20.7%) and among children from displaced families (19.6%). Urban children demonstrated a higher prevalence of anxiety and depressive symptoms compared to their rural counterparts (13.8% vs. 9.4%). No marked differences by sex were observed.

Table D1. Percentage of school-age children’s mental health status by sex and sociodemographic characteristics, 2022-2025

| Categories | Mental health status | | | | | | χ2 | p |
| --- | --- | --- | --- | --- | --- | --- | --- | --- |
|  | normal | | | anxiety and depression signs | | |  |  |
|  | n | % | SE^1^ | n | % | SE |  |  |
| **Year:** |  |  |  |  |  |  |  |  |
| 2022 | 384 | 75.3 | 1.9 | 126 | 24.7 | 1.9 | 164.4 | 0.001 |
| 2023 | 1210 | 80.8 | 1.0 | 288 | 19.2 | 1.0 |  |  |
| 2024 | 1211 | 88.3 | 0.9 | 160 | 11.7 | 0.9 |  |  |
| 2025 | 3779 | 90.6 | 0.5 | 393 | 9.4 | 0.5 |  |  |
| 2022-2025 | 6584 | 87.2 | 0.4 | 967 | 12.8 | 0.4 |  |  |
| **Sex:** |  |  |  |  |  |  |  |  |
| Boys | 3361 | 87.5 | 0.5 | 481 | 12.5 | 0.5 | 0.6 | 0.447 |
| Girls | 3223 | 86.9 | 0.6 | 486 | 13.1 | 0.6 |  |  |
| **Age group:** |  |  |  |  |  |  |  |  |
| Children | 3462 | 88.0 | 0.5 | 471 | 12.0 | 0.5 | 5.1 | 0.243 |
| Adolescents | 3122 | 86.3 | 0.6 | 496 | 13.7 | 0.6 |  |  |
| ^2^**BMI:** |  |  |  |  |  |  |  |  |
| underweight | 249 | 84.4 | 2.1 | 46 | 15.6 | 2.1 | 2.3 | 0.318 |
| normal | 4542 | 87.4 | 0.5 | 655 | 12.6 | 0.5 |  |  |
| overweight and obesity | 1784 | 87.0 | 0.7 | 266 | 13.0 | 0.7 |  |  |
| **Chronic Diseases:** |  |  |  |  |  |  |  |  |
| No | 4466 | 91.5 | 0.4 | 412 | 8.5 | 0.4 | 231.5 | 0.001 |
| Yes | 2104 | 79.3 | 0.8 | 549 | 20.7 | 0.8 |  |  |
| **Migration:** |  |  |  |  |  |  |  |  |
| Non-displaced | 4268 | 89.5 | 0.4 | 501 | 10.5 | 0.4 | 70.1 | 0.001 |
| Returned | 1461 | 84.6 | 0.9 | 266 | 15.4 | 0.9 |  |  |
| Abroad^3^ | 194 | 83.3 | 2.4 | 39 | 16.7 | 2.4 |  |  |
| IDPs^4^ | 661 | 80.4 | 1.4 | 161 | 19.6 | 1.4 |  |  |
| **Place of residence:** |  |  |  |  |  |  |  |  |
| Urban | 4988 | 86.2 | 0.5 | 800 | 13.8 | 0.5 | 23.2 | 0.001 |
| Rural | 1593 | 90.6 | 0.7 | 166 | 9.4 | 0.7 |  |  |
| **Parental education levels:** |  |  |  |  |  |  |  |  |
| Higher education | 4407 | 86.5 | 0.5 | 686 | 13.5 | 0.5 | 7.2 | 0.028 |
| Vocational education | 1497 | 88.2 | 0.8 | 201 | 11.8 | 0.8 |  |  |
| General secondary education | 676 | 89.5 | 1.1 | 79 | 10.5 | 1.1 |  |  |
| Notes:  ^1^SE is standard error of the proportion.  ^2^BMI is body mass index.  ^3^Austria, Bulgaria, Great Britain, Georgia, Denmark, Estonia, Israel, Ireland, Spain, Italy, Kazakhstan, Canada, Kuwait, Latvia, Lithuania, Moldova, Netherlands, Germany, Norway, Poland, Portugal, Romania, Slovakia, USA, Turkey, Hungary, Finland, France, Croatia, Czech Republic, Montenegro, Switzerland, Sweden  ^4^IDPs are Internally Displaced Persons | | | | | | | | |

Table D2 presents help-seeking behavior and perceived helpfulness of psychological support, stratified by symptom presence and child sex.

During 2023–2024, 21.9% of parents whose children screened positive for anxiety or depression reported contacting a mental health professional, compared to 7.7% in the group without such symptoms (χ² = 280.2; p < 0.001).

Among children with elevated symptoms, 53.9% of parents rated the received support as helpful, compared to 74.6% in the group without symptoms (χ² = 29.9; p < 0.001). These differences were consistently observed across both boys and girls.

Within the group of children with elevated symptoms, no significant variation in help-seeking or perceived helpfulness was found by age, chronic illness status, BMI category, migration experience, place of residence, or parental education level (p > 0.1).

Table D2. Percentage of school-age children who received necessary treatment or counseling, categorized by sex

| Categories | Mental  health | Boys | | | χ^2^ | p | Girls | | | χ^2^ | p |
| --- | --- | --- | --- | --- | --- | --- | --- | --- | --- | --- | --- |
|  |  |  |  |  |  |  |  |  |  |  |  |
|  |  | n | % | SE |  |  | n | % | SE |  |  |
| Consultation with  a mental health  professional,  2022-2025 | normal | 259 | 51.1 | 2.2 | 140.7 | 0.001 | 248 | 48.9 | 2.2 | 139.5 | 0.001 |
|  | anxiety and  depression  sings | 120 | 49.8 | 3.2 |  |  | 121 | 50.2 | 3.2 |  |  |
| Perceived  helpfulness of  psychological  support,  2023-2025 | normal | 191 | 53.4 | 2.6 | 13.4 | 0.001 | 167 | 46.7 | 2.6 | 16.7 | 0.001 |
|  | anxiety and  depression  sings | 66 | 55.5 | 4.6 |  |  | 53 | 44.5 | 4.6 |  |  |

Table D3 presents the “sought” stage of the four-step care cascade (perceived need → sought → received → perceived helpfulness). It captures the proportion of parents who reported contacting a psychologist, psychotherapist, or psychiatrist for their child’s psychological well-being.

During 2022–2025, 9.9% of respondents reported seeking psychological counseling for their children. All families who sought help reported receiving it, while 0.2% consulted somatic specialists instead (e.g., neurologists or family doctors).

Help-seeking increased significantly over time, from 6.9% in 2022 to 10.2% in 2025 (p < 0.01), suggesting rising awareness and/or service availability. No significant differences were observed by child sex (p > 0.9) or BMI (p > 0.7). However, migration status, place of residence, parental education, presence of chronic illness, and child age group were all significantly associated with help-seeking behavior (all p < 0.05).

Younger children (ages 8–11) were more likely to receive psychological support than adolescents, which may reflect greater parental oversight or more active referral pathways in primary schools..

Table D3. Percentage of school-age children who received psychological counseling, 2022-2025

| Categories | Consultation with  a mental health professional | | | | | | χ^2^ | p |
| --- | --- | --- | --- | --- | --- | --- | --- | --- |
|  | No | | | Yes | | |  |  |
|  | n | % | SE^1^ | n | % | SE |  |  |
| **Year:** |  |  |  |  |  |  |  |  |
| 2022 | 475 | 93.1 | 1.1 | 35 | 6.9 | 1.1 | 13.2 | 0.004 |
| 2023 | 1370 | 91.5 | 0.7 | 128 | 8.5 | 0.7 |  |  |
| 2024 | 1212 | 88.4 | 0.9 | 159 | 11.6 | 0.9 |  |  |
| 2025 | 3746 | 89.8 | 0.5 | 426 | 10.2 | 0.5 |  |  |
| 2022-2025 | 6803 | 90.1 | 0.3 | 748 | 9.9 | 0.3 |  |  |
| **Sex:** |  |  |  |  |  |  |  |  |
| Boys | 3463 | 90.1 | 0.5 | 379 | 9.9 | 0.5 | 0.01 | 0.903 |
| Girls | 3340 | 90.1 | 0.5 | 369 | 9.9 | 0.5 |  |  |
| **Age group:** |  |  |  |  |  |  |  |  |
| Children | 3514 | 89.3 | 0.5 | 419 | 10.7 | 0.5 | 5.1 | 0.023 |
| Adolescents | 3289 | 90.9 | 0.5 | 329 | 9.1 | 0.5 |  |  |
| **BMI^2^:** |  |  |  |  |  |  |  |  |
| underweight | 262 | 88.8 | 1.8 | 33 | 11.2 | 1.8 | 0.7 | 0.719 |
| normal | 4688 | 90.2 | 0.4 | 509 | 9.8 | 0.4 |  |  |
| overweight and obesity | 1844 | 90.0 | 0.7 | 206 | 10.0 | 0.7 |  |  |
| **Chronic Diseases:** |  |  |  |  |  |  |  |  |
| No | 4531 | 92.9 | 0.4 | 347 | 7.1 | 0.4 | 120.9 | 0.001 |
| Yes | 2254 | 85.0 | 0.7 | 399 | 15.0 | 0.7 |  |  |
| **Migration:** |  |  |  |  |  |  |  |  |
| Non-displaced | 4420 | 92.7 | 0.4 | 349 | 7.3 | 0.4 | 118.9 | 0.001 |
| Returned | 1515 | 87.7 | 0.8 | 212 | 12.3 | 0.8 |  |  |
| Abroad^3^ | 192 | 82.4 | 2.5 | 41 | 17.6 | 2.5 |  |  |
| IDPs^4^ | 676 | 82.2 | 1.3 | 146 | 17.8 | 1.3 |  |  |
| **Place of residence:** |  |  |  |  |  |  |  |  |
| Urban | 5137 | 88.8 | 0.4 | 651 | 11.2 | 0.4 | 49.7 | 0.001 |
| Rural | 1662 | 94.5 | 0.5 | 97 | 5.5 | 0.5 |  |  |
| **Parental education levels:** |  |  |  |  |  |  |  |  |
| Higher education | 4549 | 89.3 | 0.4 | 544 | 10.7 | 0.4 | 10.4 | 0.006 |
| Vocational education | 1556 | 91.6 | 0.7 | 142 | 8.4 | 0.7 |  |  |
| General secondary education | 693 | 91.8 | 1.0 | 62 | 8.2 | 1.0 |  |  |
| **Anxiety and depression signs** |  |  |  |  |  |  |  |  |
| No | 6077 | 92.3 | 0.3 | 507 | 7.7 | 0.3 | 280.2 | 0.001 |
| Yes | 726 | 75.1 | 1.4 | 241 | 24.9 | 1.4 |  |  |
| Notes: ^1^SE is standard error of the proportion.  ^2^BMI is body mass index.  ^3^Austria, Bulgaria, Great Britain, Georgia, Denmark, Estonia, Israel, Ireland, Spain, Italy, Kazakhstan, Canada, Kuwait, Latvia, Lithuania, Moldova, Netherlands, Germany, Norway, Poland, Portugal, Romania, Slovakia, USA, Turkey, Hungary, Finland, France, Croatia, Czech Republic, Montenegro, Switzerland, Sweden  ^4^IDPs are Internally Displaced Persons | | | | | | | | |

Table D4 presents the final stage of the psychosocial support cascade—perceived helpfulness, as reported by parents who had previously sought support for their children.

Between 2023 and 2025, 68.1% of parents who accessed mental health services rated the psychological support their child received as helpful. This rate did not significantly vary by child’s sex, age, year of survey, place of residence, or migration status (p > 0.1), suggesting overall consistency across sociodemographic groups.

However, perceived helpfulness was significantly lower among children with elevated mental health needs. Only 53.9% of parents of children exhibiting anxiety and depressive symptoms considered the received support helpful, compared to 74.6% among parents of children without these symptoms (χ² = 29.9; p < 0.001).

A similar reduction in perceived helpfulness was observed for children with chronic illnesses, highlighting a potential gap in responsiveness for those with more complex or clinical symptom profiles.

Table D4. Percentage of school-age children who received helpful psychological assistance, 2023-2025

| Categories | Perceived helpfulness of psychological assistance | | | | | | χ^2^ | p |
| --- | --- | --- | --- | --- | --- | --- | --- | --- |
|  | No | | | Yes | | |  |  |
|  | n | % | SE | n | % | SE |  |  |
| **Year:** |  |  |  |  |  |  |  |  |
| 2022 |  |  |  |  |  |  |  |  |
| 2023 | 49 | 40.2 | 4.4 | 73 | 59.8 | 4.4 | 5.2 | 0.161 |
| 2024 | 50 | 32.7 | 3.8 | 103 | 67.3 | 3.8 |  |  |
| 2025 | 125 | 29.3 | 2.2 | 301 | 70.7 | 2.2 |  |  |
| 2022-2025 | 224 | 31.9 | 1.8 | 477 | 68.1 | 1.8 |  |  |
| **Sex:** |  |  |  |  |  |  |  |  |
| Boys | 106 | 29.2 | 2.4 | 257 | 70.8 | 2.4 | 2.6 | 0.105 |
| Girls | 118 | 34.9 | 2.6 | 220 | 65.1 | 2.6 |  |  |
| **Age group:** |  |  |  |  |  |  |  |  |
| Children | 122 | 30.8 | 2.3 | 274 | 69.2 | 2.3 | 0.6 | 0.458 |
| Adolescents | 102 | 33.4 | 2.7 | 203 | 66.6 | 2.7 |  |  |
| **BMI:** |  |  |  |  |  |  |  |  |
| underweight | 6 | 21.4 | 7.8 | 22 | 78.6 | 7.8 | 1.7 | 0.426 |
| normal | 159 | 32.9 | 2.1 | 324 | 67.1 | 2.1 |  |  |
| overweight and obesity | 59 | 31.1 | 3.4 | 131 | 69.0 | 3.4 |  |  |
| **Chronic Diseases:** |  |  |  |  |  |  |  |  |
| No | 89 | 27.8 | 2.5 | 231 | 72.2 | 2.5 | 4.9 | 0.028 |
| Yes | 135 | 35.6 | 2.5 | 244 | 64.4 | 2.5 |  |  |
| **Migration:** |  |  |  |  |  |  |  |  |
| Non-displaced | 100 | 31.2 | 2.6 | 220 | 68.8 | 2.6 | 2.1 | 0.559 |
| Returned | 72 | 34.6 | 3.3 | 136 | 65.4 | 3.3 |  |  |
| Abroad | 14 | 36.8 | 7.8 | 24 | 63.2 | 7.8 |  |  |
| IDPs | 38 | 28.1 | 3.9 | 97 | 71.9 | 3.9 |  |  |
| **Place of residence:** |  |  |  |  |  |  |  |  |
| Urban | 202 | 33.2 | 1.9 | 407 | 66.8 | 1.9 | 3.2 | 0.076 |
| Rural | 22 | 23.9 | 4.4 | 70 | 76.1 | 4.4 |  |  |
| **Parental education levels:** |  |  |  |  |  |  |  |  |
| Higher education | 166 | 32.8 | 2.1 | 340 | 67.2 | 2.1 | 4.1 | 0.132 |
| Vocational education | 46 | 33.8 | 4.1 | 90 | 66.2 | 4.1 |  |  |
| General secondary education | 12 | 20.3 | 5.2 | 47 | 79.7 | 5.2 |  |  |
| **Anxiety and depression signs** |  |  |  |  |  |  |  |  |
| No | 122 | 25.4 | 2.0 | 358 | 74.6 | 2.0 | 29.9 | 0.001 |
| Yes | 102 | 46.1 | 3.4 | 119 | 53.9 | 3.4 |  |  |

Tables D5–D8: Subgroup-Specific Weighted Proportions Along the Service Cascade (2023–2025) These tables present weighted estimates of key cascade indicators — perceived need, help-seeking, receipt of support, and perceived helpfulness — across demographic and psychosocial subgroups. Post-stratification weights were applied by sex, place of residence, and educational stage (Appendix B). Table D5 summarizes demographic gradients across the cascade (e.g., age, sex, residence). Table D6 focuses on children with chronic illnesses, highlighting lower perceived helpfulness among health-vulnerable groups. Table D7 presents gradients by migration status and rural vs. urban residence. Table D8 explores how parental mental health (PHQ-2 and GAD-2) relates to access and perceptions of psychological support for children. Together, these subgroup analyses reveal key inequalities in access and satisfaction across vulnerability dimensions.

Table D5. Subgroup-Specific Weighted Proportions Along the Service Cascade: Demographic gradients, 2023-2025

| Cascade | Strata | Unweighted | | | | Weighted | | | |
| --- | --- | --- | --- | --- | --- | --- | --- | --- | --- |
|  |  | n | % | m | N | Estimated n | % | m | Estimated N |
| need | All | 737 | 12.1 | 0.4 | 6080 | 778 | 12.0 | 0.4 | 6489 |
| received | All | 713 | 10.1 | 0.3 | 7041 | 754 | 9.9 | 0.3 | 7581 |
| helpful | All | 477 | 68.0 | 1.7 | 701 | 504 | 68.1 | 1.7 | 740 |
| need | Boys | 382 | 12.3 | 0.6 | 3106 | 402 | 12.1 | 0.6 | 3323 |
| received | Boys | 368 | 10.2 | 0.5 | 3592 | 271 | 7.0 | 0.4 | 3883 |
| helpful | Boys | 257 | 70.8 | 2.3 | 363 | 271 | 70.9 | 2.3 | 382 |
| need | Girls | 355 | 11.9 | 0.6 | 2974 | 376 | 11.9 | 0.6 | 3166 |
| received | Girls | 345 | 10.0 | 0.5 | 3449 | 366 | 9.9 | 0.5 | 3698 |
| helpful | Girls | 220 | 65.1 | 2.5 | 338 | 233 | 65.1 | 2.5 | 358 |
| need | Children | 421 | 13.0 | 0.6 | 3246 | 432 | 12.7 | 0.6 | 3406 |
| received | Children | 404 | 10.8 | 0.5 | 3725 | 414 | 10.5 | 0.5 | 3941 |
| helpful | Children | 274 | 69.2 | 2.3 | 396 | 281 | 69.4 | 2.3 | 405 |
| need | Adolescents | 316 | 11.2 | 0.6 | 2834 | 346 | 11.2 | 0.6 | 3083 |
| received | Adolescents | 309 | 9.3 | 0.5 | 3316 | 340 | 9.3 | 0.5 | 3640 |
| helpful | Adolescents | 203 | 66.6 | 2.6 | 305 | 223 | 66.6 | 2.6 | 335 |

Notes. Weighted using post-stratification weights for sex, place of residence, and educational stage. See Appendix B for weighting procedures. Estimated n and N reflect weighted case frequencies.

Table D6. Subgroup-Specific Weighted Proportions Along the Service Cascade: Health-related vulnerability, 2023-2025

| Cascade | Strata | Categories | Unweighted | | | | Weighted | | | |
| --- | --- | --- | --- | --- | --- | --- | --- | --- | --- | --- |
|  |  |  | n | % | m | N | Estimated n | % | m | Estimated N |
| need | Chronic physical conditions | No | 338 | 8.6 | 0.4 | 3940 | 353 | 8.4 | 0.4 | 4183 |
| received | Chronic physical conditions | No | 325 | 7.1 | 0.4 | 4551 | 340 | 7.0 | 0.4 | 4872 |
| helpful | Chronic physical conditions | No | 231 | 72.2 | 2.5 | 320 | 242 | 72.5 | 2.4 | 334 |
| need | Chronic physical conditions | Yes | 396 | 18.6 | 0.8 | 2133 | 421 | 18.3 | 0.8 | 2297 |
| received | Chronic physical conditions | Yes | 386 | 15.6 | 0.7 | 2470 | 411 | 15.3 | 0.7 | 2685 |
| helpful | Chronic physical conditions | Yes | 244 | 64.4 | 2.4 | 379 | 259 | 64.3 | 2.4 | 403 |
| need | Mental health symptoms | No | 504 | 9.3 | 0.4 | 5431 | 528 | 9.1 | 0.4 | 5782 |
| received | Mental health symptoms | No | 491 | 7.9 | 0.3 | 6209 | 514 | 7.7 | 0.3 | 6666 |
| helpful | Mental health symptoms | No | 360 | 74.5 | 1.9 | 483 | 379 | 75.0 | 1.9 | 505 |
| need | Mental health symptoms | Yes | 233 | 35.9 | 1.8 | 649 | 250 | 35.4 | 1.8 | 707 |
| received | Mental health symptoms | Yes | 222 | 26.7 | 1.5 | 832 | 240 | 26.2 | 1.5 | 915 |
| helpful | Mental health symptoms | Yes | 117 | 53.7 | 3.3 | 218 | 125 | 53.2 | 3.3 | 235 |

Notes. Weighted using post-stratification weights for sex, place of residence, and educational stage. See Appendix B for weighting procedures. Estimated n and N reflect weighted case frequencies.

Table D7. Subgroup-Specific Weighted Proportions Along the Service Cascade: Migration & place of residence, 2023-2025

| Cascade | Strata | Unweighted | | | | Weighted | | | |
| --- | --- | --- | --- | --- | --- | --- | --- | --- | --- |
|  |  | n | % | m | N | Estimated n | % | m | Estimated N |
| need | Urban | 641 | 14.2 | 0.5 | 4508 | 652 | 14.2 | 0.5 | 4598 |
| received | Urban | 619 | 11.6 | 0.4 | 5348 | 630 | 11.6 | 0.4 | 5438 |
| helpful | Urban | 407 | 66.8 | 1.9 | 609 | 413 | 66.6 | 1.9 | 620 |
| need | Rural | 96 | 6.1 | 0.6 | 1570 | 126 | 6.7 | 0.6 | 1888 |
| received | Rural | 94 | 5.6 | 0.5 | 1689 | 124 | 5.8 | 0.5 | 2137 |
| helpful | Rural | 70 | 76.1 | 3.9 | 92 | 91 | 75.8 | 3.9 | 120 |
| need | Non-displaced | 341 | 8.4 | 0.4 | 4064 | 368 | 8.4 | 0.4 | 4369 |
| received | Non-displaced | 326 | 7.4 | 0.4 | 4432 | 353 | 7.3 | 0.4 | 4804 |
| helpful | Non-displaced | 220 | 68.8 | 2.5 | 320 | 239 | 69.1 | 2.5 | 346 |
| need | Returned | 219 | 16.5 | 1.0 | 1329 | 225 | 16.2 | 1.0 | 1393 |
| received | Returned | 212 | 12.3 | 0.8 | 1727 | 218 | 12.0 | 0.8 | 1817 |
| helpful | Returned | 136 | 65.4 | 3.3 | 208 | 140 | 65.7 | 3.3 | 213 |
| need | Abroad | 39 | 32.5 | 4.1 | 120 | 40 | 30.1 | 4.0 | 133 |
| received | Abroad | 38 | 18.7 | 2.6 | 203 | 39 | 17.1 | 2.5 | 228 |
| helpful | Abroad | 24 | 63.2 | 7.7 | 38 | 24 | 61.5 | 7.8 | 39 |
| need | IDPs | 138 | 24.3 | 1.8 | 567 | 145 | 24.4 | 1.8 | 594 |
| received | IDPs | 137 | 20.2 | 1.5 | 679 | 144 | 19.7 | 1.5 | 732 |
| helpful | IDPs | 97 | 71.9 | 3.8 | 135 | 101 | 71.1 | 3.8 | 142 |

Notes. Weighted using post-stratification weights for sex, place of residence, and educational stage. See Appendix B for weighting procedures. Estimated n and N reflect weighted case frequencies.

Table D8. Subgroup-Specific Weighted Proportions Along the Service Cascade: Parents` mental health, 2023-2025

| Cascade | Strata | Categories | Unweighted | | | | Weighted | | | |
| --- | --- | --- | --- | --- | --- | --- | --- | --- | --- | --- |
|  |  |  | n | % | m | N | Estimated n | % | m | Estimated N |
| need | Parental anxiety | No | 405 | 9.4 | 0.4 | 4294 | 437 | 9.6 | 0.4 | 4565 |
| received | Parental anxiety | No | 393 | 8.2 | 0.4 | 4819 | 424 | 8.2 | 0.4 | 5165 |
| helpful | Parental anxiety | No | 289 | 74.9 | 2.1 | 386 | 310 | 74.5 | 2.1 | 416 |
| need | Parental anxiety | Yes | 273 | 18.8 | 1.0 | 1450 | 300 | 19.1 | 1.0 | 1570 |
| received | Parental anxiety | Yes | 267 | 15.0 | 0.8 | 1777 | 292 | 15.1 | 0.8 | 1936 |
| helpful | Parental anxiety | Yes | 160 | 61.1 | 2.9 | 262 | 175 | 61.0 | 2.9 | 287 |
| need | Parental depression | No | 378 | 9.4 | 0.4 | 4032 | 412 | 9.6 | 0.4 | 4292 |
| received | Parental depression | No | 371 | 8.2 | 0.4 | 4508 | 404 | 8.3 | 0.4 | 4842 |
| helpful | Parental depression | No | 277 | 75.7 | 2.2 | 366 | 300 | 75.4 | 2.2 | 398 |
| need | Parental depression | Yes | 300 | 17.5 | 0.9 | 1712 | 325 | 17.6 | 0.9 | 1843 |
| received | Parental depression | Yes | 289 | 13.8 | 0.7 | 2087 | 312 | 13.8 | 0.7 | 2259 |
| helpful | Parental depression | Yes | 172 | 61.0 | 2.8 | 282 | 185 | 60.7 | 2.8 | 305 |

Notes. Weighted using post-stratification weights for sex, place of residence, and educational stage. See Appendix B for weighting procedures. Estimated n and N reflect weighted case frequencies.

Table D9. Barriers to Accessing Psychological Support (Open-Ended Responses), 2023-2025

Open-ended responses from parents who reported trauma exposure but did not seek psychological support were thematically coded into six categories. The most frequent barriers included reliance on self-help, concerns about online formats, and lack of accessible in-person services. These qualitative findings complement the quantitative cascade by illuminating reasons behind unmet need.

| Barrier Category | Code | Brief Description | Frequency (n=61) | Percentage (%) | Illustrative Quotes |
| --- | --- | --- | --- | --- | --- |
| Self-help / Professional self-sufficiency | 1 | Psychologist in family, pedagogical background | 11 | 18.03 | “Mom is the best psychologist”, “I’m a psychologist”, “We talk often, solve things ourselves”, “There are psychologists in my family and I have a teaching degree” |
| Alternative support (e.g., neurologist) | 2 | Consulted non-psychologist professionals | 22 | 36.07 | “Consulted a neurologist”, “Family doctor”, “Neuropathologist”, “Consulted the family physician” |
| Lack of specialist / Inaccessibility | 3 | No available psychologist nearby or affordable, avoidance of online format or lack of in-person care | 7 | 11.48 | “Couldn’t find a suitable psychologist”, “Haven’t consulted yet, need to find a good one”, “Too expensive”, “Would like to, but can’t afford it”, “Psychologist only available online, but we prefer in person”, “Psychologist willing to help only online” |
| Planning / Not yet initiated | 4 | Intended but not yet implemented | 7 | 11.48 | “We’re preparing to”, “Signed up”, “Scheduled for summer”, “Currently looking for a psychologist” |
| Child refusal | 5 | Child refuses or avoids participation | 4 | 6.56 | “Refuses”, “Afraid”, “Doesn’t want to”, “Doesn’t want to see a psychologist” |
| Other / Unclear | 6 | Difficult to categorize | 10 | 16.39 | “No possibility”, “No”, “Not sure how”, “Partially” |

Table D10. Social support levels, 2022-2025

Across all examined subgroups, parents consistently rated support from their immediate social environment (friends, family, volunteers) as higher than support from state institutions (e.g., local authorities, social services). This gap was especially pronounced among internally displaced families, those living abroad, and parents of children with anxiety and depressive symptoms. Notably, perceived state support remained low (mean scores ~2.0/5) even among groups with higher psychosocial vulnerability, underscoring persistent gaps in public MHPSS outreach and perceived institutional responsiveness.

| Categories | Support of social group,  2022-2025 | | Social support of state authorities,  2024-2025 | |
| --- | --- | --- | --- | --- |
| **Year** | Means | SD | Means | SD |
| 2022 | 3.5 | 1.2 |  |  |
| 2023 | 3.6 | 1.2 |  |  |
| 2024 | 3.5 | 1.2 | 1.8 | 1.1 |
| 2025 | 3.6 | 1.1 | 2.0 | 1.2 |
| Total | 3.6 | 1.2 | 2.0 | 1.1 |
| F/p | 6.9/0.001 | | 18.4/0.001 | |
| **Migration:** |  |  |  |  |
| Non-displaced | 3.6 | 1.2 | 2.0 | 1.1 |
| Returned | 3.6 | 1.1 | 2.0 | 1.1 |
| Abroad | 3.8 | 1.2 | 2.3 | 1.4 |
| IDPs | 3.4 | 1.2 | 2.2 | 1.2 |
| F/p | 5.5/0.001 | | 4.8/0.003 | |
| **Place of residence:** |  |  |  |  |
| Urban | 3.6 | 1.2 | 2.0 | 1.1 |
| Rural | 3.5 | 1.2 | 2.1 | 1.2 |
| F/p | 7.5/0.006 | | 9.9/0.002 | |
| **Age group:** |  |  |  |  |
| Children | 3.6 | 1.1 | 2.0 | 1.2 |
| Adolescents | 3.5 | 1.2 | 2.0 | 1.1 |
| F/p | 21.3/0.001 | | 1.7/0.192 | |
| **Sex:** |  |  |  |  |
| Boys | 3.6 | 1.2 | 2.0 | 1.2 |
| Girls | 3.6 | 1.2 | 2.0 | 1.1 |
| F/p | 0.5/0.478 | | 0.2/0.651 | |
| **Chronic Diseases:** |  |  |  |  |
| No | 3.6 | 1.1 | 2.0 | 1,2 |
| Yes | 3.5 | 1.2 | 1.9 | 1,1 |
| F/p | 31.9/0.001 | | 8.6/0.003 | |
| **Anxiety and depression signs** |  |  |  |  |
| No | 3.6 | 1.1 | 2.0 | 1,2 |
| Yes | 3.1 | 1.2 | 1.8 | 1,1 |
| F/p | 149.1/0.001 | | 18.3/0.001 | |
| **Parental education levels:** |  |  |  |  |
| Higher education | 3.6 | 1.1 | 2.0 | 1,1 |
| Vocational education | 3.5 | 1.2 | 1.9 | 1,2 |
| General secondary education | 3.5 | 1.3 | 2.2 | 1,3 |
| F/p | 3.7/0.023 | | 8.4/0.001 | |

# **Appendix E. Stratified associations between social support and perceived helpfulness of psychological support**

Table E1. Perceived helpfulness of psychological support (%) by social support level across migration status, health status, and place of residence, 2023-2025

| Categories | Social Support | Perceived helpfulness | | | | | | Row totals | χ^2^ | p |
| --- | --- | --- | --- | --- | --- | --- | --- | --- | --- | --- |
|  |  | No | | | Yes | | |  |  |  |
|  |  | n | % | m | n | % | m |  |  |  |
|  |  | Migration | | | | | |  |  |  |
| Non-displaced | Low | 26 | 41.9 | 6.3 | 36 | 58.1 | 6.3 | 62 | 8.1 | 0.004 |
| Non-displaced | High | 71 | 27.8 | 2.8 | 184 | 72.2 | 2.8 | 255 |  |  |
| Non-displaced | Total | 97 | 30.6 | 2.6 | 220 | 69.4 | 2.6 | 317 |  |  |
| Returned | Low | 21 | 51.2 | 7.8 | 20 | 48.8 | 7.8 | 41 | 4.3 | 0.037 |
| Returned | High | 47 | 29.2 | 3.6 | 114 | 70.8 | 3.6 | 161 |  |  |
| Returned | Total | 68 | 33.7 | 3.3 | 134 | 66.3 | 3.3 | 202 |  |  |
| Abroad | Low | 3 | 60.0 | 21.9 | 2 | 40.0 | 21.9 | 5 | 4.6 | 0.032 |
| Abroad | High | 9 | 32.1 | 8.8 | 19 | 67.9 | 8.8 | 28 |  |  |
| Abroad | Total | 12 | 36.4 | 8.4 | 21 | 63.6 | 8.4 | 33 |  |  |
| IDPs | Low | 12 | 41.4 | 9.1 | 17 | 58.6 | 9.1 | 29 | 16.6 | 0.001 |
| IDPs | High | 26 | 24.5 | 4.2 | 80 | 75.5 | 4.2 | 106 |  |  |
| IDPs | Total | 38 | 28.1 | 3.9 | 97 | 71.9 | 3.9 | 135 |  |  |
|  |  | Chronic Diseases | | | | | |  |  |  |
| No | Low | 22 | 42.3 | 6.9 | 30 | 57.7 | 6.9 | 52 | 5.3 | 0.021 |
| No | High | 64 | 24.2 | 2.6 | 200 | 75.8 | 2.6 | 264 |  |  |
| No | Total | 86 | 27.2 | 2.5 | 230 | 72.8 | 2.5 | 316 |  |  |
| Yes | Low | 40 | 47.1 | 5.4 | 45 | 52.9 | 5.4 | 85 | 13.8 | 0.001 |
| Yes | High | 89 | 31.1 | 2.7 | 197 | 68.9 | 2.7 | 286 |  |  |
| Yes | Total | 129 | 34.8 | 2.5 | 242 | 65.2 | 2.5 | 371 |  |  |
|  |  | Place of residence | | | | | | |  |  |
| Urban | Low | 118 | 40.5 | 2.9 | 173 | 59.5 | 2.9 | 291 | 16.6 | 0.005 |
| Urban | High | 76 | 24.9 | 2.5 | 229 | 75.1 | 2.5 | 305 |  |  |
| Urban | Total | 194 | 32.6 | 1.9 | 402 | 67.4 | 1.9 | 596 |  |  |
| Rural | Low | 14 | 31.1 | 6.9 | 31 | 68.9 | 6.9 | 45 | 3.3 | 0.070 |
| Rural | High | 7 | 15.2 | 5.3 | 39 | 84.8 | 5.3 | 46 |  |  |
| Rural | Total | 21 | 23.1 | 4.4 | 70 | 76.9 | 4.4 | 91 |  |  |
|  |  | Anxiety and depression signs | | | | | | |  |  |
| No | Low | 67 | 32.4 | 3.3 | 140 | 67.6 | 3.3 | 207 | 10.6 | 0.001 |
| No | High | 52 | 19.3 | 2.4 | 217 | 80.7 | 2.4 | 269 |  |  |
| No | Total | 119 | 25.0 | 2.0 | 357 | 75.0 | 2.0 | 476 |  |  |
| Yes | Low | 65 | 50.4 | 4.4 | 64 | 49.6 | 4.4 | 129 | 3.2 | 0.073 |
| Yes | High | 31 | 37.8 | 5.4 | 51 | 62.2 | 5.4 | 82 |  |  |
| Yes | Total | 96 | 45.5 | 3.4 | 115 | 54.5 | 3.4 | 211 |  |  |

**Note,** The table presents row percentages of parents reporting psychological support as helpful (“Yes”) versus not helpful (“No”), stratified by social support level (low vs, high) and key subgroups (migration status, chronic illness, place of residence, and presence of anxiety/depressive symptoms),
Low social support includes scores 1–3; high social support includes scores 4–5,
Pearson’s chi-square test was used where expected cell counts were sufficient; likelihood-ratio (M–L) chi-square statistics were applied when expected counts were <10,

# **Appendix F. Exploratory logistic regression models: interaction terms and weighted analyses**

This appendix presents exploratory logistic regression models designed to evaluate the robustness of the main findings under varying analytical conditions. The models include interaction terms and apply post-stratification weights based on child sex, school education stage (primary, middle, secondary), and place of residence (urban vs rural). All models were estimated using the same predictor set and inclusion criteria as the main analyses.

Table F1. Multicollinearity diagnostics for predictors included in the adjusted logistic regression models,

| Model | Collinearity Statistics | | |
| --- | --- | --- | --- |
|  | Predictors | Tolerance | VIF |
| 1 | Migr | 0.969 | 1.032 |
|  | Region | 0.917 | 1.090 |
|  | Place | 0.877 | 1.140 |
|  | AgeGr | 0.937 | 1.067 |
|  | Sex | 0.967 | 1.034 |
|  | ChD | 0.953 | 1.050 |
|  | Edu | 0.947 | 1.056 |
|  | SocP | 0.878 | 1.140 |
|  | MH2Gr | 0.818 | 1.222 |
|  | PsyE | 0.916 | 1.092 |
|  | AnxP | 0.667 | 1.499 |
|  | DeprP | 0.653 | 1.532 |

Note: Variable abbreviations correspond to those used in the regression models and are defined in the Methods section

Table F2. Adjusted logistic regression model for help-seeking (excluding 2022; no interaction terms)

| **Variables in the Equation** | | | | | | | | | |
| --- | --- | --- | --- | --- | --- | --- | --- | --- | --- |
| Model | | B | S.E. | Wald | df | Sig. | Exp(B) | 95% C.I.for EXP(B) | |
|  |  |  |  |  |  |  |  | Lower | Upper |
| Step 1 | year |  |  | 34.249 | 2 | 0.000 |  |  |  |
|  | year(1) | -0.770 | 0.140 | 30.407 | 1 | 0.000 | 0.463 | 0.352 | 0.609 |
|  | year(2) | 0.015 | 0.111 | 0.018 | 1 | 0.893 | 1.015 | 0.816 | 1.263 |
|  | Migr |  |  | 87.514 | 3 | 0.000 |  |  |  |
|  | Migr(1) | -1.107 | 0.121 | 83.026 | 1 | 0.000 | 0.331 | 0.261 | 0.419 |
|  | Migr(2) | -0.774 | 0.136 | 32.278 | 1 | 0.000 | 0.461 | 0.353 | 0.602 |
|  | Migr(3) | -0.190 | 0.259 | 0.537 | 1 | 0.464 | 0.827 | 0.498 | 1.374 |
|  | Region1 | 0.042 | 0.046 | 0.863 | 1 | 0.353 | 1.043 | 0.954 | 1.141 |
|  | Place | -0.550 | 0.118 | 21.831 | 1 | 0.000 | 0.577 | 0.458 | 0.727 |
|  | AgeGr | -0.248 | 0.094 | 6.983 | 1 | 0.008 | 0.780 | 0.649 | 0.938 |
|  | Sex | -0.007 | 0.087 | 0.006 | 1 | 0.939 | 0.993 | 0.838 | 1.178 |
|  | BMI | 0.008 | 0.013 | 0.420 | 1 | 0.517 | 1.008 | 0.983 | 1.034 |
|  | ChD | 0.642 | 0.089 | 52.123 | 1 | 0.000 | 1.899 | 1.596 | 2.261 |
|  | Edu |  |  | 1.563 | 2 | 0.458 |  |  |  |
|  | Edu(1) | -0.051 | 0.155 | 0.107 | 1 | 0.743 | 0.951 | 0.702 | 1.287 |
|  | Edu(2) | -0.175 | 0.173 | 1.024 | 1 | 0.312 | 0.840 | 0.598 | 1.178 |
|  | SocP | 0.004 | 0.038 | 0.014 | 1 | 0.907 | 1.004 | 0.932 | 1.083 |
|  | MH2Gr | 1.246 | 0.109 | 130.203 | 1 | 0.000 | 3.477 | 2.807 | 4.308 |
|  | AnxP | 0.209 | 0.109 | 3.648 | 1 | 0.056 | 1.232 | 0.995 | 1.526 |
|  | DeprP | 0.114 | 0.106 | 1.138 | 1 | 0.286 | 1.120 | 0.909 | 1.380 |
|  | Constant | -1.813 | 0.354 | 26.267 | 1 | 0.000 | 0.163 |  |  |

Table F3. Adjusted logistic regression model for help-seeking (2022–2025; with interaction terms)

| **Variables in the Equation** | | | | | | | | | |
| --- | --- | --- | --- | --- | --- | --- | --- | --- | --- |
| Model | | B | S,E, | Wald | df | Sig, | Exp(B) | 95% C,I,for EXP(B) | |
|  |  |  |  |  |  |  |  | Lower | Upper |
| Step 2 | year |  |  | 40.679 | 3 | 0.000 |  |  |  |
|  | year(1) | -1.078 | 0.372 | 8.408 | 1 | 0.004 | 0.340 | 0.164 | 0.705 |
|  | year(2) | -0.776 | 0.139 | 31.174 | 1 | 0.000 | 0.460 | 0.351 | 0.604 |
|  | year(3) | 0.011 | 0.111 | 0.010 | 1 | 0.920 | 1.011 | 0.813 | 1.257 |
|  | Migr |  |  | 73.423 | 3 | 0.000 |  |  |  |
|  | Migr(1) | -1.103 | 0.133 | 68.692 | 1 | 0.000 | 0.332 | 0.256 | 0.431 |
|  | Migr(2) | -0.767 | 0.140 | 29.916 | 1 | 0.000 | 0.465 | 0.353 | 0.611 |
|  | Migr(3) | -0.128 | 0.248 | 0.264 | 1 | 0.607 | 0.880 | 0.541 | 1.432 |
|  | Region1 | 0.049 | 0.045 | 1.198 | 1 | 0.274 | 1.051 | 0.962 | 1.148 |
|  | Place | -0.520 | 0.134 | 14.976 | 1 | 0.000 | 0.595 | 0.457 | 0.774 |
|  | AgeGr | -0.241 | 0.093 | 6.672 | 1 | 0.010 | 0.786 | 0.655 | 0.944 |
|  | Sex | -0.004 | 0.086 | 0.002 | 1 | 0.967 | 0.996 | 0.841 | 1.180 |
|  | BMI | 0.010 | 0.013 | 0.645 | 1 | 0.422 | 1.010 | 0.985 | 1.036 |
|  | ChD | 0.644 | 0.088 | 53.309 | 1 | 0.000 | 1.904 | 1.602 | 2.264 |
|  | Edu |  |  | 1.552 | 2 | 0.460 |  |  |  |
|  | Edu(1) | -0.086 | 0.152 | 0.322 | 1 | 0.571 | 0.917 | 0.681 | 1.236 |
|  | Edu(2) | -0.194 | 0.170 | 1.299 | 1 | 0.254 | 0.824 | 0.591 | 1.149 |
|  | SocP | 0.022 | 0.044 | 0.251 | 1 | 0.616 | 1.023 | 0.937 | 1.116 |
|  | MH2Gr | 1.442 | 0.289 | 24.935 | 1 | 0.000 | 4.229 | 2.401 | 7.447 |
|  | AnxP | 0.217 | 0.109 | 3.984 | 1 | 0.046 | 1.242 | 1.004 | 1.536 |
|  | DeprP | 0.105 | 0.106 | 0.989 | 1 | 0.320 | 1.111 | 0.903 | 1.367 |
|  | migration × place of residence | -0.019 | 0.099 | 0.039 | 1 | 0.844 | 0.981 | 0.809 | 1.190 |
|  | symptoms × social support | -0.069 | 0.082 | 0.696 | 1 | 0.404 | 0.934 | 0.794 | 1.097 |
|  | Constant | -1.911 | 0.362 | 27.841 | 1 | 0.000 | 0.148 |  |  |

Table F4. Adjusted logistic regression model for perceived helpfulness (2023–2025; with interaction terms)

| **Variables in the Equation** | | | | | | | | | |
| --- | --- | --- | --- | --- | --- | --- | --- | --- | --- |
| Model | | B | S.E. | Wald | df | Sig. | Exp(B) | 95% C.I.for EXP(B) | |
|  |  |  |  |  |  |  |  | Lower | Upper |
| Step 3 | year |  |  | 1.209 | 2 | 0.546 |  |  |  |
|  | year(1) | -0.316 | 0.287 | 1.207 | 1 | 0.272 | 0.729 | 0.415 | 1.281 |
|  | year(2) | -0.070 | 0.235 | 0.088 | 1 | 0.767 | 0.933 | 0.588 | 1.479 |
|  | Migr |  |  | 0.176 | 3 | 0.981 |  |  |  |
|  | Migr(1) | -0.047 | 0.264 | 0.032 | 1 | 0.859 | 0.954 | 0.569 | 1.601 |
|  | Migr(2) | -0.113 | 0.282 | 0.160 | 1 | 0.689 | 0.893 | 0.514 | 1.552 |
|  | Migr(3) | -0.121 | 0.511 | 0.056 | 1 | 0.813 | 0.886 | 0.325 | 2.414 |
|  | Region1 | -0.021 | 0.093 | 0.050 | 1 | 0.823 | 0.979 | 0.817 | 1.174 |
|  | Place | 0.295 | 0.307 | 0.926 | 1 | 0.336 | 1.343 | 0.736 | 2.450 |
|  | AgeGr | 0.107 | 0.200 | 0.286 | 1 | 0.593 | 1.113 | 0.752 | 1.646 |
|  | Sex | -0.374 | 0.185 | 4.096 | 1 | 0.043 | 0.688 | 0.479 | 0.988 |
|  | BMI | -0.029 | 0.027 | 1.144 | 1 | 0.285 | 0.972 | 0.922 | 1.024 |
|  | ChD | -0.339 | 0.188 | 3.251 | 1 | 0.071 | 0.713 | 0.493 | 1.030 |
|  | Edu |  |  | 2.337 | 2 | 0.311 |  |  |  |
|  | Edu(1) | -0.545 | 0.382 | 2.033 | 1 | 0.154 | 0.580 | 0.274 | 1.227 |
|  | Edu(2) | -0.620 | 0.414 | 2.239 | 1 | 0.135 | 0.538 | 0.239 | 1.212 |
|  | SocP | 0.341 | 0.105 | 10.605 | 1 | 0.001 | 1.407 | 1.146 | 1.727 |
|  | MH2Gr | 0.006 | 0.557 | 0.000 | 1 | 0.991 | 1.006 | 0.338 | 2.997 |
|  | AnxP | -0.216 | 0.221 | 0.963 | 1 | 0.327 | 0.805 | 0.523 | 1.241 |
|  | DeprP | -0.209 | 0.222 | 0.887 | 1 | 0.346 | 0.811 | 0.525 | 1.254 |
|  | migration × place of residence | 0.141 | 0.248 | 0.325 | 1 | 0.568 | 1.152 | 0.709 | 1.872 |
|  | symptoms × social support | -0.176 | 0.162 | 1.184 | 1 | 0.277 | 0.838 | 0.610 | 1.152 |
|  | Constant | 1.554 | 0.804 | 3.737 | 1 | 0.053 | 4.729 |  |  |

Table F5. Weighted logistic regression model (sex weight)

| **Variables in the Equation** | | | | | | | | | |
| --- | --- | --- | --- | --- | --- | --- | --- | --- | --- |
| Model |  | B | S.E. | Wald | df | Sig. | Exp(B) | 95% C.I.for EXP(B) | |
|  |  |  |  |  |  |  |  | Lower | Upper |
| Step 4 | year |  |  | 0.873 | 2 | 0.646 |  |  |  |
|  | year(1) | -0.255 | 0.274 | 0.866 | 1 | 0.352 | 0.775 | 0.453 | 1.326 |
|  | year(2) | -0.091 | 0.229 | 0.158 | 1 | 0.691 | 0.913 | 0.583 | 1.430 |
|  | Migr |  |  | 0.342 | 3 | 0.952 |  |  |  |
|  | Migr(1) | -0.104 | 0.243 | 0.184 | 1 | 0.668 | 0.901 | 0.560 | 1.450 |
|  | Migr(2) | -0.146 | 0.269 | 0.296 | 1 | 0.587 | 0.864 | 0.509 | 1.465 |
|  | Migr(3) | -0.196 | 0.482 | 0.166 | 1 | 0.684 | 0.822 | 0.320 | 2.113 |
|  | Region1 | -0.005 | 0.093 | 0.003 | 1 | 0.957 | 0.995 | 0.829 | 1.194 |
|  | Place | 0.406 | 0.300 | 1.831 | 1 | 0.176 | 1.501 | 0.834 | 2.701 |
|  | Sex | -0.343 | 0.178 | 3.705 | 1 | 0.054 | 0.710 | 0.501 | 1.006 |
|  | BMI | -0.025 | 0.025 | 1.019 | 1 | 0.313 | 0.975 | 0.929 | 1.024 |
|  | ChD | -0.228 | 0.182 | 1.576 | 1 | 0.209 | 0.796 | 0.557 | 1.137 |
|  | Edu |  |  | 2.025 | 2 | 0.363 |  |  |  |
|  | Edu(1) | -0.495 | 0.375 | 1.737 | 1 | 0.188 | 0.610 | 0.292 | 1.272 |
|  | Edu(2) | -0.570 | 0.408 | 1.951 | 1 | 0.163 | 0.566 | 0.254 | 1.258 |
|  | SocP | 0.269 | 0.079 | 11.460 | 1 | 0.001 | 1.309 | 1.120 | 1.529 |
|  | AnxP | -0.311 | 0.216 | 2.075 | 1 | 0.150 | 0.733 | 0.480 | 1.119 |
|  | DeprP | -0.130 | 0.218 | 0.358 | 1 | 0.550 | 0.878 | 0.573 | 1.345 |
|  | MH2Gr | -0.596 | 0.198 | 9.036 | 1 | 0.003 | 0.551 | 0.373 | 0.813 |
|  | Constant | 1.657 | 0.754 | 4.834 | 1 | 0.028 | 5.245 |  |  |

Table F6. Weighted logistic regression model (education weight)

| **Variables in the Equation** | | | | | | | | | |
| --- | --- | --- | --- | --- | --- | --- | --- | --- | --- |
| Model |  | B | S.E. | Wald | df | Sig. | Exp(B) | 95% C.I.for EXP(B) | |
|  |  |  |  |  |  |  |  | Lower | Upper |
| Step 5 | year |  |  | 0.499 | 2 | 0.779 |  |  |  |
|  | year(1) | -0.191 | 0.274 | 0.485 | 1 | 0.486 | 0.826 | 0.483 | 1.413 |
|  | year(2) | -0.080 | 0.227 | 0.124 | 1 | 0.724 | 0.923 | 0.592 | 1.440 |
|  | Migr |  |  | 0.313 | 3 | 0.958 |  |  |  |
|  | Migr(1) | -0.055 | 0.242 | 0.052 | 1 | 0.820 | 0.946 | 0.588 | 1.522 |
|  | Migr(2) | -0.113 | 0.270 | 0.174 | 1 | 0.677 | 0.893 | 0.526 | 1.517 |
|  | Migr(3) | -0.241 | 0.474 | 0.259 | 1 | 0.611 | 0.786 | 0.310 | 1.991 |
|  | Region1 | 0.005 | 0.093 | 0.003 | 1 | 0.958 | 1.005 | 0.837 | 1.206 |
|  | Place | 0.424 | 0.301 | 1.980 | 1 | 0.159 | 1.528 | 0.846 | 2.759 |
|  | Sex | -0.315 | 0.177 | 3.155 | 1 | 0.076 | 0.730 | 0.515 | 1.033 |
|  | BMI | -0.024 | 0.025 | 0.931 | 1 | 0.335 | 0.976 | 0.930 | 1.025 |
|  | ChD | -0.179 | 0.182 | 0.965 | 1 | 0.326 | 0.836 | 0.585 | 1.195 |
|  | Edu |  |  | 2.356 | 2 | 0.308 |  |  |  |
|  | Edu(1) | -0.490 | 0.378 | 1.680 | 1 | 0.195 | 0.613 | 0.292 | 1.285 |
|  | Edu(2) | -0.629 | 0.410 | 2.356 | 1 | 0.125 | 0.533 | 0.239 | 1.190 |
|  | SocP | 0.258 | 0.079 | 10.600 | 1 | 0.001 | 1.295 | 1.108 | 1.513 |
|  | AnxP | -0.209 | 0.214 | 0.958 | 1 | 0.328 | 0.811 | 0.533 | 1.234 |
|  | DeprP | -0.140 | 0.217 | 0.415 | 1 | 0.520 | 0.870 | 0.568 | 1.330 |
|  | MH2Gr | -0.728 | 0.197 | 13.680 | 1 | 0.000 | 0.483 | 0.328 | 0.710 |
|  | Constant | 1.548 | 0.752 | 4.239 | 1 | 0.040 | 4.702 |  |  |

Table F7. Weighted logistic regression model (place of residence)

| **Variables in the Equation** | | | | | | | | | |
| --- | --- | --- | --- | --- | --- | --- | --- | --- | --- |
|  | | | | | | | | | |
| Model |  | B | S.E. | Wald | df | Sig. | Exp(B) | 95% C.I.for EXP(B) | |
|  |  |  |  |  |  |  |  | Lower | Upper |
| Step 6 | year |  |  | 1.104 | 2 | 0.576 |  |  |  |
|  | year(1) | -0.298 | 0.285 | 1.096 | 1 | 0.295 | 0.742 | 0.425 | 1.297 |
|  | year(2) | -0.057 | 0.235 | 0.059 | 1 | 0.808 | 0.944 | 0.596 | 1.496 |
|  | Migr |  |  | 0.271 | 3 | 0.965 |  |  |  |
|  | Migr(1) | -0.114 | 0.245 | 0.217 | 1 | 0.641 | 0.892 | 0.552 | 1.443 |
|  | Migr(2) | -0.125 | 0.277 | 0.205 | 1 | 0.651 | 0.882 | 0.513 | 1.517 |
|  | Migr(3) | -0.150 | 0.508 | 0.088 | 1 | 0.767 | 0.860 | 0.318 | 2.329 |
|  | Region1 | -0.026 | 0.093 | 0.077 | 1 | 0.781 | 0.975 | 0.813 | 1.168 |
|  | Place | 0.388 | 0.272 | 2.033 | 1 | 0.154 | 1.474 | 0.865 | 2.512 |
|  | Sex | -0.348 | 0.181 | 3.692 | 1 | 0.055 | 0.706 | 0.495 | 1.007 |
|  | BMI | -0.024 | 0.025 | 0.938 | 1 | 0.333 | 0.976 | 0.929 | 1.025 |
|  | ChD | -0.314 | 0.185 | 2.870 | 1 | 0.090 | 0.730 | 0.508 | 1.051 |
| Step 7 | Edu |  |  | 2.405 | 2 | 0.300 |  |  |  |
|  | Edu(1) | -0.559 | 0.381 | 2.153 | 1 | 0.142 | 0.572 | 0.271 | 1.206 |
|  | Edu(2) | -0.621 | 0.413 | 2.260 | 1 | 0.133 | 0.538 | 0.239 | 1.207 |
|  | SocP | 0.272 | 0.081 | 11.184 | 1 | 0.001 | 1.312 | 1.119 | 1.538 |
|  | AnxP | -0.228 | 0.218 | 1.094 | 1 | 0.296 | 0.796 | 0.519 | 1.221 |
|  | DeprP | -0.196 | 0.222 | 0.780 | 1 | 0.377 | 0.822 | 0.532 | 1.270 |
|  | MH2Gr | -0.558 | 0.202 | 7.650 | 1 | 0.006 | 0.572 | 0.385 | 0.850 |
|  | Constant | 1.789 | 0.765 | 5.471 | 1 | 0.019 | 5.981 |  |  |

Table F8. Weighted logistic regression model (total weight)

| **Variables in the Equation** | | | | | | | | | |
| --- | --- | --- | --- | --- | --- | --- | --- | --- | --- |
| Model |  | B | S.E. | Wald | df | Sig. | Exp(B) | 95% C.I.for EXP(B) | |
|  |  |  |  |  |  |  |  | Lower | Upper |
| Step 8 | year |  |  | 0.634 | 2 | 0.728 |  |  |  |
|  | year(1) | -0.225 | 0.284 | 0.625 | 1 | 0.429 | 0.799 | 0.457 | 1.394 |
|  | year(2) | -0.038 | 0.233 | 0.027 | 1 | 0.869 | 0.962 | 0.609 | 1.521 |
|  | Migr |  |  | 0.207 | 3 | 0.976 |  |  |  |
|  | Migr(1) | -0.076 | 0.246 | 0.095 | 1 | 0.758 | 0.927 | 0.572 | 1.501 |
|  | Migr(2) | -0.098 | 0.278 | 0.124 | 1 | 0.724 | 0.907 | 0.526 | 1.563 |
|  | Migr(3) | -0.198 | 0.501 | 0.157 | 1 | 0.692 | 0.820 | 0.307 | 2.188 |
|  | Region1 | -0.017 | 0.093 | 0.035 | 1 | 0.852 | 0.983 | 0.819 | 1.179 |
|  | Place | 0.423 | 0.274 | 2.377 | 1 | 0.123 | 1.526 | 0.892 | 2.613 |
|  | Sex | -0.325 | 0.181 | 3.231 | 1 | 0.072 | 0.722 | 0.507 | 1.030 |
|  | BMI | -0.023 | 0.025 | 0.833 | 1 | 0.362 | 0.977 | 0.930 | 1.027 |
|  | ChD | -0.267 | 0.186 | 2.075 | 1 | 0.150 | 0.765 | 0.532 | 1.101 |
|  | Edu |  |  | 2.527 | 2 | 0.283 |  |  |  |
|  | Edu(1) | -0.543 | 0.384 | 2.000 | 1 | 0.157 | 0.581 | 0.274 | 1.233 |
|  | Edu(2) | -0.657 | 0.415 | 2.504 | 1 | 0.114 | 0.518 | 0.230 | 1.170 |
|  | SocP | 0.263 | 0.081 | 10.503 | 1 | 0.001 | 1.300 | 1.109 | 1.524 |
|  | AnxP | -0.131 | 0.217 | 0.363 | 1 | 0.547 | 0.877 | 0.573 | 1.343 |
|  | DeprP | -0.198 | 0.222 | 0.798 | 1 | 0.372 | 0.820 | 0.530 | 1.267 |
|  | MH2Gr | -0.687 | 0.200 | 11.741 | 1 | 0.001 | 0.503 | 0.340 | 0.745 |
|  | Constant | 1.666 | 0.764 | 4.755 | 1 | 0.029 | 5.292 |  |  |

1. Yelizarova O.T., Hozak S.V., Stankevich T.V., Parats A.M., Yelizarov V.O. Validity and reliability assessment of the RCADS-P-25 in the Ukrainian population. Environment & Health. 2024. 3 (112): 10-16. <https://doi.org/10.32402/dovkil2024.03.010> [↑](#footnote-ref-1)
